# Spatial and Temporal Trends of Persistent Organic Pollutants across Europe after 15 Years of MONET Passive Air Sampling

**DOI:** 10.1021/acs.est.3c00796

**Published:** 2023-07-26

**Authors:** Kevin B. White, Jiří Kalina, Martin Scheringer, Petra Přibylová, Petr Kukučka, Jiří Kohoutek, Roman Prokeš, Jana Klánová

**Affiliations:** † RECETOX, Masaryk University, 625 00 Brno, Czech Republic; ‡ Institute of Biogeochemistry and Pollutant Dynamics, ETH Zürich, 8092 Zürich, Switzerland

**Keywords:** air pollution, passive sampling, POPs, Stockholm Convention, trend analysis

## Abstract

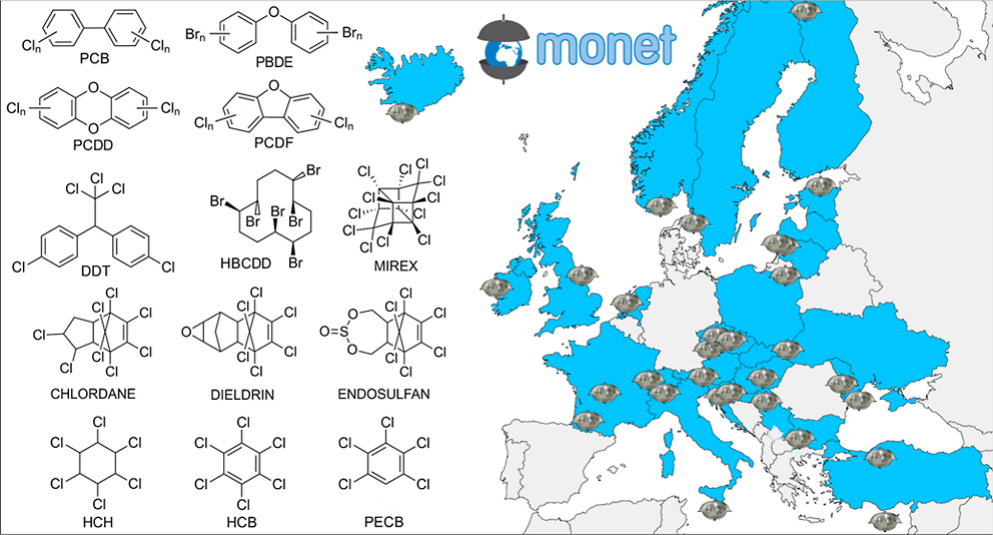

The Global Monitoring Plan of the Stockholm Convention on Persistent Organic Pollutants (POPs) was established to generate long-term data necessary for evaluating the effectiveness of regulatory measures at a global scale. After 15 years of passive air monitoring (2003–2019), MONET is the first network to produce sufficient data for the analysis of continuous long-term temporal trends of POPs in air across the entire European continent. This study reports long-term concentrations of 20 POPs monitored at 32 sites in 27 European countries. As of January 1, 2019, the concentration ranges (pg/m^3^) were 1.1–52.8 (∑_6_PCB), 0.3–8.5 (∑_12_dl-PCB), 0.007–0.175 (∑_17_PCDD/F), 0.02–2.2 (∑_9_PBDE), 0.4–24.7 (BDE 209), 0.5–247 (∑_6_DDT), 1.7–818 (∑_4_HCH), 15.8–74.7 (HCB), and 5.9–21.5 (PeCB). Temporal trends indicate that concentrations of most POPs have declined significantly over the past 15 years, with median annual decreases ranging from −8.0 to −11.5% (halving times of 6–8 years) for ∑_6_PCB, ∑_17_PCDD/F, HCB, PeCB, and ∑_9_PBDE. Furthermore, no statistically significant differences were observed in either the trends or the concentrations of specific POPs at sites in Western Europe (WEOG) compared to sites in Central and Eastern Europe (CEE), which suggests relatively uniform compound-specific distribution and removal at the continental scale.

## Abstract 2

Long-term temporal trends of persistent organic pollutants in air were determined for the first time across the entire European continent in support of effectiveness evaluation of the Stockholm Convention.

## Introduction

The Stockholm Convention on Persistent Organic Pollutants (POPs) entered into force in 2004 with the aim of protecting human health and the environment by reducing or eliminating the production and release of these compounds. To evaluate the effectiveness of regulatory measures and assess long-term trends, a Global Monitoring Plan was established to provide a harmonized framework for the collection of comparable POP monitoring data in air across countries within the five United Nations Regional Groups. Within Europe, the countries fall into two Regional Groups based primarily on geographic divisions, and as a result, implementation of the Stockholm Convention and monitoring of POPs in air across Europe is addressed separately by the “Western Europe and Others Group” (WEOG) and the “Central and Eastern Europe Group” (CEE). Due to differences in the production, use, and regulation of these compounds, CEE countries historically had higher atmospheric burdens of certain POPs compared to WEOG countries, particularly organochlorine pesticides (OCPs).^1,2^

Initial monitoring of POPs in European air began in the early 1990s using active air samplers under the European Monitoring and Evaluation Programme (EMEP) (Norwegian Institute for Air Research, NILU), though monitoring of conventional air pollutants had already been ongoing at EMEP stations across Europe since the early 1970s.^3^ Continental air sampling campaigns for POPs occurred in 2006 at 86 EMEP stations in 34 countries^1^ and in 2016 at 101 EMEP stations in 33 countries.^100^ However, continuous long-term monitoring of POPs (>15 years) is ongoing at only 6 EMEP stations: Birkenes (Norway), Košetice (Czech Republic), Pallas (Finland), Råö (Sweden), Stórhöfdi (Iceland), and Zeppelin (Svalbard, Norway), as well as three more recent sites in Germany (Waldhof, Westerland, and Zingst). Except for Košetice, these monitoring stations are all located in WEOG countries, with three (Pallas, Stórhöfdi, and Zeppelin) also part of the global Arctic Monitoring and Assessment Programme (AMAP).^4,5^ In addition to EMEP, the Toxic Organic Micro Pollutants (TOMPs) network (Lancaster University, United Kingdom) has also been monitoring POPs in air since the early 1990s at six sites in England and Scotland.^6^ Since EMEP and TOMPs are the longest-operating POP monitoring networks in Europe, studies of long-term temporal POP trends in European air have almost exclusively focused on Northern/Western Europe and the Arctic,^3−12^ where the conditions may not reflect those of the rest of the continent.

Apart from long-term active air monitoring of POPs at Košetice since 1996,^13^ monitoring data outside of the WEOG region were rather limited prior to the Stockholm Convention. The first significant atmospheric monitoring campaign for POPs in CEE countries was APOPSBAL, a European Union Framework Program project investigating the extent to which residents of the former Yugoslavia were exposed to elevated POP levels following the Balkan wars. In 2003–2004, RECETOX (Masaryk University, CZ) coordinated active and passive air sampling campaigns of POPs at 34 sampling sites across Bosnia and Herzegovina, Croatia, and Serbia^14,15^ and at 18 reference passive air sampling sites across the Czech Republic.^15^ These 18 Czech sampling sites formed the basis for the establishment of the MONET passive air sampling network, which continues to monitor the long-term atmospheric burden of POPs across the Czech Republic.^16,17^ In 2004, the Global Atmospheric Passive Sampling (GAPS) network (Environment Canada) was also established to monitor POPs and other airborne contaminants at sites around the world;^18−20^ however, ongoing long-term GAPS monitoring in Europe occurs primarily at sites in WEOG countries.^21−23^ Thus, following the conclusion of the APOPSBAL campaign, routine MONET passive sampling expanded into an additional 18 CEE countries in 2006–2008^2^ and then expanded again into an additional 14 WEOG countries in 2009 to generate consistent and comparable long-term air monitoring data for the entire continent.

Compared to the active air samplers used by EMEP and TOMPs, passive samplers used by MONET and GAPS are cheap and do not require electricity, which made them ideal for capacity building across Europe, particularly in the CEE region.^24^ We have previously reported comparable atmospheric trends^13,25^ and concentrations^26,27^ of POPs between MONET passive samplers and co-located active samplers; thus, the MONET passive sampling network is a valuable tool in locations where long-term active sampling is challenging or unfeasible. Although there have been numerous independent and short-term air sampling studies of POPs at individual sites or in specific countries over the last two decades, there have been few attempts to generate consistent long-term air monitoring data in Europe at sites outside of the major networks (EMEP/TOMPs/GAPS/MONET). The two exceptions are an international active sampling network in alpine regions of Austria, Germany, Italy, Slovenia, and Switzerland (MONARPOP)^28,29^ and a national Spanish passive air sampling network.^30,31^ MONET is the largest POP monitoring network in Europe, with 32 long-term monitoring sites (all >7 years) in 27 countries across the continent (in addition to 26 other long-term monitoring sites just within the Czech Republic). As a result, this is the first study to report continuous long-term temporal trends of atmospheric concentrations of legacy POPs across the entire European continent as well as the first study to report atmospheric concentrations of some “new” Stockholm Convention POPs in countries within the CEE region. Given the uniquely high number of sites and large geographic coverage of the MONET network in Europe, we also performed cluster and spatial analyses to assess whether any geographic trends of POP concentrations in air could be identified across the continent.

## Methods

### Monitoring Sites

This study follows a methodology similar to our recent assessment of long-term temporal trends of atmospheric POP concentrations at MONET passive sampling sites across Africa.^32^ MONET passive sampling sites across Europe with at least five years of continuous monitoring data were selected for this study, most with data as of 2019 and some with data as of 2017/2018. Two exceptions were made for the sites at Lahemaa (EE) and Starina (SK), both of which stopped monitoring in 2015 but had data since 2006 (9.1 and 9.7 years, respectively). As a result, long-term temporal trends of atmospheric POP concentrations were calculated at 32 sites in 27 countries (all with >7 years of monitoring), with an equal number of sites in both WEOG and CEE countries (sites and country codes are listed in Table 1). Apart from Çamkoru (TR), Fruška Gora (RS), Plateliai (LT), and Zagreb (HR), all MONET sites included in this study are located at EMEP air monitoring stations, including the six stations with long-term EMEP POP data (Birkenes, Košetice, Pallas, Råö, Stórhöfd̵i, and Zeppelin). As a result, most sites included in this study are classified as background sites and are rural or remote and distant from major population centers. Although there are an additional 26 MONET sites with long-term POP monitoring in the Czech Republic alone,^16,17^ only the four MONET sites located at Czech EMEP stations (Churáňov, Košetice, Prague Libuš, and Svratouch) were included here so as not to bias the analysis toward the results of a single country. All MONET passive air sampling data reported here are freely accessible online through the Genasis Database^33^ hosted by RECETOX. Inactive and short-term MONET sites in Europe that were excluded from this study are listed in Table S1 in the Supporting Information (SI-1).

**Table 1:** MONET Passive Air Sampling Sites across Europe Included in This Study^a^

UN region	country	site name	code	latitude	longitude	type	monitoring	years	samples
WEOG	Austria	Sonnblick^c^	AT	47.054	12.958	remote	2009–2017	7.6	28
WEOG^b^	Cyprus	Agia Marina^c^	CY	35.038	33.058	rural	2009–2019	9.3	38
WEOG	Finland	Pallas^d^	FI	68.000	24.246	polar	2009–2018	8.4	31
WEOG	France	Montfranc^c^	FR1	45.810	2.060	rural	2009–2019	9.5	37
WEOG	France	Peyrusse-Vieille^c^	FR2	43.630	0.180	rural	2009–2019	9.4	28
WEOG	Iceland	Stórhöfdi^d^	IS	63.400	–20.283	remote	2009–2018	8.9	35
WEOG	Ireland	Mace Head^c^	IE	53.330	–9.900	rural	2009–2017	8.0	33
WEOG	Italy	Ispra^c^	IT	45.817	8.633	rural	2009–2018	8.8	23
WEOG	Malta	Ġordan Lighthouse^c^	MT	36.073	14.219	rural	2009–2019	9.6	40
WEOG	Netherlands	De Zilk^c^	NL	52.297	4.511	rural	2009–2019	9.7	41
WEOG	Norway	Birkenes^d^	NO1	58.383	8.250	remote	2009–2019	9.8	39
WEOG	Norway	Zeppelin^d^	NO2	78.880	11.883	polar	2009–2019	9.8	29
WEOG	Sweden	Råö^d^	SE	57.394	11.914	remote	2009–2019	9.5	39
WEOG	Switzerland	Payerne^c^	CH	46.800	6.933	rural	2009–2019	9.6	40
WEOG	Turkey	Çamkoru	TR	40.585	32.505	rural	2009–2018	8.6	37
WEOG	United Kingdom	High Muffles^c^	UK	54.140	–0.460	rural	2009–2019	9.4	36
CEE	Bulgaria	Moussala^c^	BG	42.179	23.585	remote	2009–2019	9.8	40
CEE	Croatia	Zagreb	HR	45.836	15.983	urban	2004–2019	14.4	31
CEE	Czech Republic	Churáňov^c^	CZ1	49.068	13.615	remote	2006–2019	13.0	61
CEE	Czech Republic	Košetice^d^	CZ2	49.573	15.080	rural	2003–2019	15.8	70
CEE	Czech Republic	Prague Libuš^c^	CZ3	50.007	14.446	suburban	2004–2019	15.0	67
CEE	Czech Republic	Svratouch^c^	CZ4	49.735	16.034	rural	2006–2019	12.8	58
CEE	Estonia	Lahemaa^c^	EE	59.515	25.928	remote	2006–2015	9.1	26
CEE	Hungary	K-puszta^c^	HU	46.968	19.553	rural	2009–2019	9.3	34
CEE	Latvia	Rucava^c^	LV	56.162	21.173	rural	2006–2019	12.8	40
CEE	Lithuania	Plateliai	LT	56.010	21.887	rural	2006–2019	12.7	42
CEE	Moldova	Leova^c^	MD	46.500	28.300	rural	2007–2017	9.8	22
CEE	Poland	Diabla Góra^c^	PL	54.125	22.038	rural	2009–2019	9.4	39
CEE	Serbia	Fruška Gora	RS	45.159	19.863	remote	2004–2019	14.4	45
CEE	Slovakia	Starina^c^	SK	49.043	22.260	rural	2006–2015	9.7	30
CEE	Slovenia	Iskrba^c^	SI	45.561	14.863	rural	2007–2019	11.7	41
CEE	Ukraine	Zmiinyi Island^c^	UA	45.256	30.201	remote	2009–2018	9.1	32

a WEOG: Western Europe and Others Regional Group; CEE: Central and Eastern Europe Regional Group.

b Although Cyprus is a European country, it is officially part of the Asia-Pacific UN Region; it was included in this study as WEOG due to its geographic proximity to Turkey.

c Sampling sites located at EMEP air monitoring stations.

d Sampling sites located at EMEP POP air monitoring stations.

### Passive Sampling

MONET passive air samplers consist of a polyurethane foam (PUF) disk suspended between two stainless-steel domes that protect the PUF disk from sunlight and dry and wet deposition but still allow the penetration of ambient air. MONET PUF disk characteristics and sampler housing dimensions are listed in Table S2. The use and limitations of passive PUF samplers for atmospheric monitoring of semivolatile organic compounds such as POPs is described in detail elsewhere.^34^ MONET passive samplers in Europe were deployed for continuous 28-day intervals during the initial years of monitoring (2003–2011) but are now deployed for continuous 84-day intervals at all sites since July 2011. To prevent the early samples with threefold frequency from skewing the trend analysis compared to the later, less frequent samples, the 28-day samples were aggregated quarterly (∼91 days) by calculating their weighted arithmetic mean with the concentrations weighted by the number of days of each sample in the quarter. These early aggregated samples were considered fully comparable with the later 84-day samples for trend analysis. The length of monitoring at each site varied depending on when it was established and some gaps in monitoring occurred at some sites. The sampling regime and availability of data for each compound group and site are depicted in Figure S1.

### Chemical Analysis

After each exposure period, PUF disks were collected and shipped to the RECETOX Trace Analytical Laboratories for analysis. Across the MONET network in Europe, 17 of the currently listed Stockholm Convention POPs are included in the continuous monitoring: aldrin, chlordane, dichlorodiphenyltrichloroethane (DDT), dieldrin, endrin, endosulfan, hexabromocyclododecane (HBCDD), hexachlorobenzene (HCB), hexachlorocyclohexanes (HCHs), heptachlor, mirex, polybrominated diphenyl ethers (PBDEs), polychlorinated biphenyls (PCBs), dioxin-like PCBs (dl-PCBs), polychlorinated dibenzo-*p*-dioxins (PCDDs), polychlorinated dibenzofurans (PCDFs), and pentachlorobenzene (PeCB). Additionally, chlordecone was monitored from 2011 to 2014 but was not detected in any sample at any site above the limit of quantification. Perfluorooctanoic acid (PFOA) and perfluorooctanesulfonic acid (PFOS) were similarly monitored from 2013 to 2015; however, PUF samplers do not efficiently capture these compounds, and so they were removed from routine monitoring. As a result, the data series were too short to calculate temporal trends for these compounds, and the reported concentrations should be interpreted with caution. It is important to note that only 26 of the 32 sites had long-term PBDE and HBCDD monitoring data, and only 12 sites had long-term dl-PCB and PCDD/F data, which limited their potential use in the spatial analyses. A summary of all POPs included in this study and the availability of MONET sampling data for each is provided in Table S3. An overview of the standard analytical methods is presented in Table S4, with detailed information on sampling, chemical analysis, and instrumental methods provided in Section 2 of the Supporting Information.

### Air Concentrations and Temporal Trends

Analysis of air concentrations and temporal trends was performed as described for MONET sites across Africa.^32^ Briefly, concentrations in each PUF disk (pg/PUF) were converted to concentrations in air (pg/m^3^) with the standard GAPS template model for calculating effective air sampling volumes of passive PUF samplers.^35^ Model input parameters specific to MONET samplers are given in Table S2. For more consistent continental-scale meteorological data, site-specific average temperatures over each sampling period were generated from the MERRA-2 model,^36^ as described previously for MONET^26,27,32^ and GAPS.^23,37^ A more complex model for calculating effective air sampling volumes has recently been developed^37^ and is now being used by GAPS.^23^ However, negligible differences have been observed in the output concentrations compared to the original model for the majority of sites globally.^23,26,27^ Therefore, model selection is not expected to significantly influence the temporal trends reported in this study. Temporal trends, in the form of both annual exponential increases/decreases (%) and halving/doubling times (*t*_1/2_), were estimated using the Theil-Sen linear regression estimator^38,39^ on log-transformed air concentration data for each combination of sampling site and compound. The 95% confidence intervals and their statistical significance were also calculated for each temporal trend using the nonparametric Mann-Kendall test. Since the lengths and end dates of the monitoring periods varied between sites, the temporal trends were used to extrapolate or interpolate air concentrations on January 1, 2019, for all sites/compounds for more consistent comparisons of time-dependent data. Furthermore, since multiple analytes were monitored in air for most POPs, these compounds are subsequently presented as sums: 6 indicator PCB congeners (∑_6_PCB); 12 dioxin-like PCB congeners (∑_12_dl-PCB); 7 dioxins (∑_7_PCDD), 10 furans (∑_10_PCDF), and all 17 homologues together (∑_17_PCDD/F); 9 PBDE congeners (∑_9_PBDE; excluding BDE 209); 3 HBCDD isomers (∑_3_HBCDD); 6 DDT analytes (∑_6_DDT); 4 HCH isomers (∑_4_HCH); 3 chlordane analytes (∑_3_chlordane); 3 endosulfan analytes (∑_3_endosulfan); 3 endrin analytes (∑_3_endrin); and 3 heptachlor analytes (∑_3_heptachlor). Individual compounds within each sum are listed in Table S5, with their frequency of detection over the monitoring period at each site listed in Table S6. More detailed information on temporal trend analysis is provided in Section 3 of the Supporting Information, including examples of trend plots (Figures S2 and S3).

### Spatial Analyses

To investigate whether there were any large-scale geographic differences in the temporal trends and modeled 1-Jan-2019 concentrations, preliminary analyses were performed for each set of data to calculate a continental transect (“splitting line”) for each POP that splits all sites geographically into the two most significantly different halves of the continent (e.g., northwestern vs. southeastern). The statistical significance of the difference between both halves was determined with the Mann–Whitney *U* test.^40^ In addition, multidimensional *k*-means cluster analyses were performed to characterize the similarity of the sites with respect to temporal trends and modeled concentrations independently of their geographic distribution, as previously demonstrated for POPs at MONET sites across the Czech Republic.^16^ Since some of the POPs were not monitored at all sites, and some were only monitored for a short period of time, only ∑_6_PCB, ∑_6_DDT, ∑_4_HCH, and HCB were included in the cluster analysis as they have the longest data series and were measured at each of the 32 sites. Each of the four compound groups was represented twice in the cluster analysis (once for its trends and once for its modeled 1-Jan-2019 concentrations), which provided a total of eight dimensions for the clustering. The method was run for 100 iterations, and then the most frequent result with 4 distinct clusters was selected.

## Results and Discussion

### Temporal Trends of Atmospheric Concentrations

**Figure 1:**
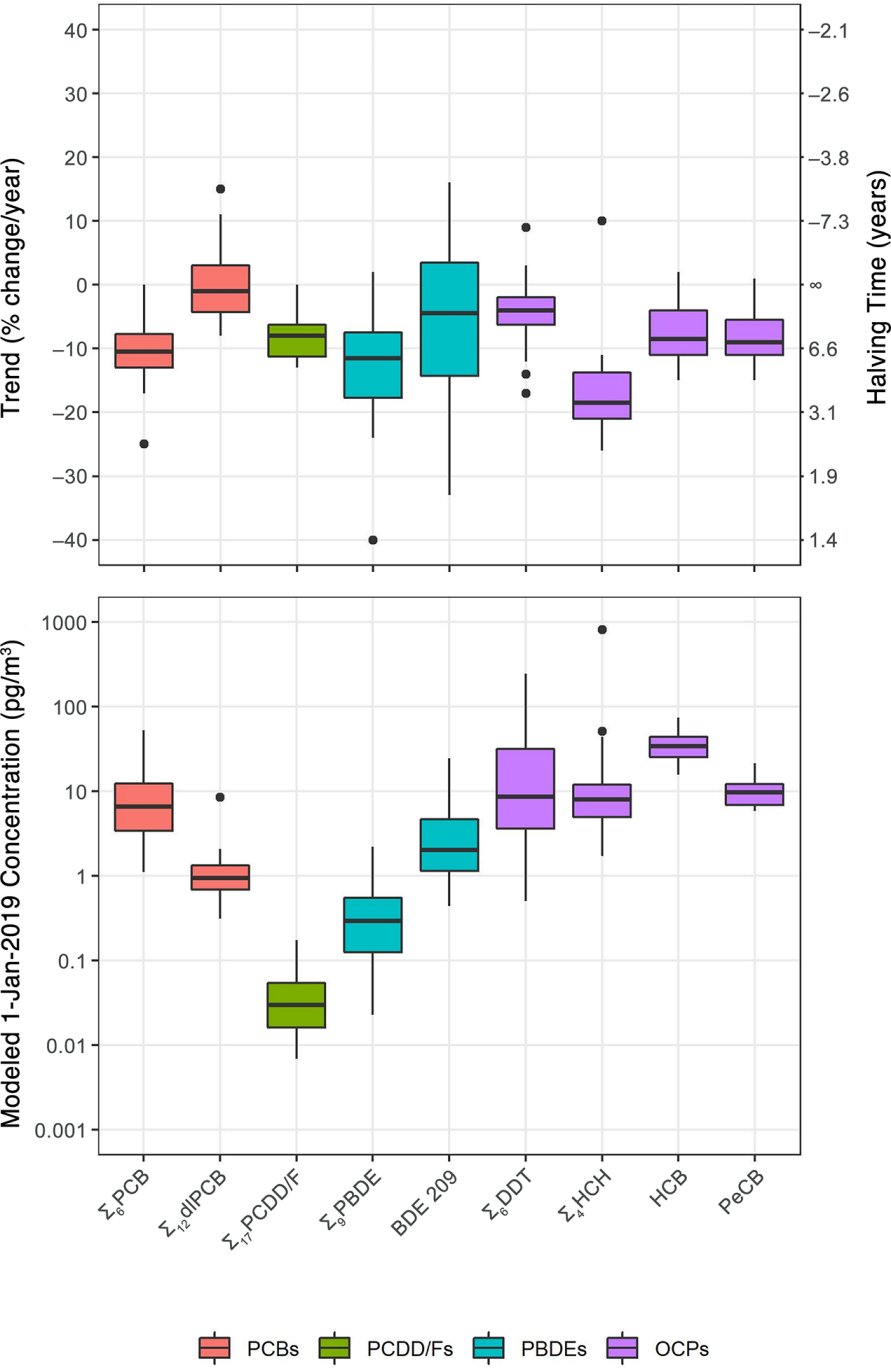
Temporal trends (% change/year and halving time; Table 2) and modeled 1-Jan-2019 concentrations (pg/m^3^; Table 3) for POPs at MONET sites across Europe with at least 7 years of continuous atmospheric monitoring data (*n* = 32 sites for ∑_6_PCB, ∑_6_DDT, ∑_4_HCH, HCB, and PECB; *n* = 26 sites for ∑_9_PBDE and BDE 209; *n* = 12 sites for ∑_12_dlPCB and ∑_17_PCDD/F). Boxes represent the 25th to 75th percentiles, with the median (50th percentile) represented by a horizontal black line. Whiskers represent the largest/smallest values no further than 1.5 × IQR from the upper/lower limits of the box; values outside of this range are considered outliers and are represented as black dots.

Temporal trends for ∑_6_PCB, ∑_12_dl-PCB, ∑_17_PCDD/F, ∑_9_PBDE, BDE 209, and OCPs (∑_6_DDT, ∑_4_HCH, HCB, and PeCB) span at least 7 years at all MONET sites (except for 6.5 years for PBDEs at Pallas) (Table 2) and were used to model atmospheric concentrations on January 1, 2019 (Table 3). These temporal trends and modeled concentrations are depicted in Figure 1. Temporal trends and modeled concentrations for the other POPs show a greater degree of uncertainty and should be interpreted with caution. Trends and concentrations for all individual compounds, as well as more detailed statistical results, are provided in the Supporting Information spreadsheet (SI-2).

**Table 2:** Temporal Trends (% Change/Year) of Atmospheric Concentrations from Continuous Monitoring Spanning at Least 7 Years^a^

site code	AT	CY	FI	FR1	FR2	IS	IE	IT	MT	NL	NO1	NO2	SE	CH	TR	UK
Polychlorinated biphenyls and Dibenzo-*p*-Dioxins/Furans																
∑_6_PCB	–25*	–16*	–13*	–9*	–6*	–12*	–14*	–9*	–8*	–3*	–7	–12	–4	–9*	–12*	0
∑_12_dl-PCB				+9						+1		+15	0		+11	
∑_17_PCDD/F				–8*						–3		–8	–4		0	
Brominated Flame Retardants																
∑_9_PBDE		–9*	–24*	–17*	–3	–9			–4	–7*	–18*	+2	0	–9*	–23*	–9*
BDE 209		–27*	+16	–12	+14	–7			–1	+11	0	–7	+2	–6	–33*	–6
Organochlorine Pesticides																
∑_6_DDT	–17	–9*	–14*	–6*	–6*	–12*	–2	–7	–2	–1	0	–14	+3	–4	–4	+9*
∑_4_HCH	–12	–18*	–21*	–14*	–12*	–23*	–13*	–19*	–22*	–14*	–19*	–12	–13*	–16*	–17*	–11*
HCB	–14*	–9*	–11*	–3	–4*	–15*	–9*	–11*	–12*	–10*	–2	–3	–5*	–6*	–10*	+2
PeCB	–12	–9*	–11*	–3	–7*	–11*	–10*	–10*	–15*	–9*	–4	–1	–3	–7	–4*	+1
																

site code	BG	HR	CZ1	CZ2	CZ3	CZ4	EE	HU	LV	LT	MD	PL	RS	SK	SI	UA
Polychlorinated Biphenyls and Dibenzo-*p*-Dioxins/Furans																
∑_6_PCB	–7	–6	–13*	–12*	–10*	–11*	–17*	–14*	–11*	–10*	–10*	–9*	–14*	–13*	–15*	–2
∑_12_dl-PCB			–4	–2	–4	–8*		0	–5				–7			
∑_17_PCDD/F			–7	–12*	–13*	–8		–12*	–8*				–11*			
Brominated Flame Retardants																
∑_9_PBDE	–13*	–24*	–20*	–3	–16*	–40*		–20*	–12	–16*		–9*	–11*		–13*	–2
BDE 209	–30*	–17	+5	+12	+4	–1		–21*	–2	+9		–6	–15		–18	–3
Organochlorine Pesticides																
∑_6_DDT	–1	–6	–6*	–1	–3*	–4*	–9*	–5	–3	–4	0	–5*	–7*	–4	–6*	–3
∑_4_HCH	–26*	+10	–19*	–15*	–17*	–18*	–24*	–21*	–23*	–19*	–20*	–21*	–19*	–19*	–22*	–11*
HCB	–8*	+2	–4*	–11*	–9*	–7*	–7	–10*	–7*	–3	–4	–12*	–14*	0	–9*	–11*
PeCB	–12*	–1	–6*	–9*	–10*	–6*	–10*	–9*	–7*	–2	–11	–13*	–11*	–3	–10*	–9*

a * denotes statistically significant trends. Refer to Table 1 for site names corresponding to each site code. Trends for individual compounds and other POPs, as well as more detailed statistical analyses (including confidence intervals and halving-times), are provided in the SI-2.

**Table 3:** Atmospheric Concentrations (pg/m^3^) as of January 1, 2019, Modeled from the Temporal Trends in Table 2^a^

site code	AT	CY	FI	FR1	FR2	IS	IE	IT	MT	NL	NO1	NO2	SE	CH	TR	UK
Polychlorinated Biphenyls and Dibenzo-*p*-Dioxins/Furans																
∑_6_PCB	2.1*	3.5*	1.1*	3.2*	4.6*	8.1*	4.0*	9.7*	18.8*	52.8*	2.2	2.3	11.3	10.3*	1.9*	6.0
∑_12_dl-PCB				0.7						8.5		0.5	1.3		0.3	
∑_17_PCDD/F				0.013*						0.175		0.007	0.031		0.013	
Brominated Flame Retardants																
∑_9_PBDE		1.3*	0.1*	0.1*	0.7	0.3			1.2	2.2*	0.1*	0.1	0.3	0.8*	0.02*	0.7*
BDE 209		22.0*	3.1	1.0	5.1	6.4			24.7	9.1	1.0	1.2	3.5	1.8	0.4*	2.1
Organochlorine Pesticides																
∑_6_DDT	1.5	20.2*	0.5*	2.0*	3.7*	6.3*	3.8	3.1	34.3	41.7	2.6	1.0	10.5	6.6	5.2	8.0*
∑_4_HCH	44.2	6.4*	1.7*	11.9*	15.8*	9.8*	10.8*	3.6*	7.9*	26.5*	3.1*	5.6	7.3*	7.5*	4.6*	13.4*
HCB	40.7*	18.4*	25.7*	35.4	24.2*	44.2*	37.3*	16.2*	15.8*	36.3*	33.9	74.7	44.6*	27.0*	18.7*	54.3
PeCB	16.0	7.6*	6.9*	10.3	6.9*	8.5*	6.6*	6.8*	5.9*	9.6*	8.6	21.5	12.7*	6.5*	6.0*	16.0

site code	BG	HR	CZ1	CZ2	CZ3	CZ4	EE	HU	LV	LT	MD	PL	RS	SK	SI	UA
Polychlorinated Biphenyls and Dibenzo-*p*-Dioxins/Furans																
∑_6_PCB	4.7	32.1	7.2*	8.5*	23.5*	14.9*	2.4*	6.1*	5.2*	4.5*	16.5*	8.4*	13.5*	12.1*	1.8*	52.0
∑12dl-PCB			0.7	0.8	2.1	1.3*		0.7	1.1				1.3			
∑17PCDD/F			0.017	0.028*	0.057*	0.069		0.053*	0.026*				0.041*			
Brominated Flame Retardants																
∑_9_PBDE	0.2*	0.3*	0.1*	0.3	0.5*	0.2*		0.2*	0.1	0.1*		0.3*	0.4*		0.1*	0.6
BDE 209	0.9*	6.4	1.1	1.5	2.0	1.2		0.7*	1.1	3.3		2.9	1.6		5.5	2.2
Organochlorine Pesticides																
∑_6_DDT	9.5	9.4	8.1*	47.1	44.2*	57.1*	2.4*	44.7	11.0	7.0	246.9	30.0*	31.2*	14.5	3.5*	222.2
∑_4_HCH	4.0*	817.9	8.3*	10.6*	9.9*	12.3*	2.0*	8.1*	3.8*	5.1*	24.8*	7.8*	10.1*	6.2*	2.8*	51.4*
HCB	44.7*	51.4	41.6*	33.8*	31.4*	52.5*	29.7	23.9*	31.1*	40.9	45.0	35.0*	23.9*	47.5	16.0*	28.6*
PeCB	12.1*	16.7	10.7*	8.8*	8.2*	11.6*	6.6*	18.6*	10.4*	14.2	9.7	8.8*	12.0*	15.0	6.5*	10.5*

a All concentrations were calculated from continuous monitoring data spanning at least 7 years (except for brominated flame retardants at FI). * denotes concentrations modeled from statistically significant trends. Refer to Table 1 for site names corresponding to each site code. Concentrations of individual compounds and other POPs, as well as more detailed statistical analyses, are provided in the SI-2.

### Polychlorinated Biphenyls

PCBs were included in the Stockholm Convention on POPs when it entered into force in 2004, but by then, they had already been restricted for several decades across Europe, with production peaking in the 1960s and then declining until the 1990s.^41,42^

A decreasing trend was observed in ∑_6_PCB concentrations at 31 of the 32 sites (25 statistically significant), with a median annual change of −10.5% corresponding to a halving time (*t*_1/2_) of 6.2 years. No change was observed at the one site without a decreasing trend (0%, High Muffles, UK). Decreasing trends are relatively uniform across the continent, with an interquartile range (IQR) of −13.0% to −7.8%. These trends correspond to a median 1-Jan-2019 ∑_6_PCB concentration of 6.6 pg/m^3^ (IQR = 3.4–12.4 pg/m^3^). Similar atmospheric trends were reported by Schuster et al., with an average *t*_1/2_ of 8.4 years for PCBs at 11 sampling sites in the UK and Norway from 1994 to 2008.^43^ Wöhrnschimmel et al.^7^ and Wong et al.^4^ found halving times of PCBs in air to typically be between 5 and 10 years at Zeppelin (NO2), consistent with our value of 5.4 years, but substantially longer halving times (around 15 years) were observed at Pallas (FI) compared to our value of 5 years.^4^ The modeled 1-Jan-2019 ∑_6_PCB concentration reported in this study at Zeppelin (2.3 pg/m^3^) was also in close agreement with the value reported by Wong et al. for the same site (∼2 pg/m^3^).^4^

### PCDD/Fs and dl-PCBs

Unlike most of the other POPs, which were intentionally produced for various applications, PCDD/Fs are unwanted by-products generated during combustion and industrial processes. Like PCBs, they were included in the Stockholm Convention in 2004.

A decreasing trend was observed in ∑_17_PCDD/F concentrations at 11 of the 12 sites (6 statistically significant), with a median annual change of −8.0% (*t*_1/2_ = 8.3 yr) and an IQR of −11.3% to −6.3%. Similar to the PCBs, no change was observed at the one site without a decreasing trend (0%, Çamkoru, TR). These trends correspond to a median 1-Jan-2019 ∑_17_PCDD/F concentration of 0.030 pg/m^3^ (IQR = 0.016–0.054 pg/m^3^). Our results are generally consistent with the shallow decreasing ∑PCDD/F trends and median concentrations (0.045–0.062 pg/m^3^) reported by Kirchner et al. at three high-altitude European atmospheric monitoring stations in Austria, Germany, and Switzerland (2008–2013/2018).^28^

Trends for dioxin-like PCBs (∑_12_dl-PCB) concentrations are much less consistent, with no clear change over time apart from a statistically significant decrease at a single site (−8%, Svratouch, CZ4). Across the 12 sites, the median annual change is −1.0%, with an IQR of −4.3% to +3.0%, indicating minimal change over time. Despite the inconsistent trends, the 1-Jan-2019 ∑_12_dl-PCB concentrations are highly consistent across all sites with a median of 1.0 pg/m^3^ and an IQR of 0.7–1.3 pg/m^3^, suggesting a relatively uniform continental background concentration.

### Polybrominated Diphenyl Ethers

PBDEs were listed in the Stockholm Convention in 2009 as the commercial penta-BDE and octa-BDE formulations (reflected in ∑_9_PBDE), but their production and use in Europe had already peaked a decade earlier in the 1990s.

A decreasing trend was observed in ∑_9_PBDE concentrations at 24 of the 26 sites (18 statistically significant), with a median annual change of −11.5% (*t*_1/2_ = 5.7 yr). Decreasing trends are similar to those for both PCBs and PCDD/Fs, with an IQR of −17.8% to −7.5%. There is one extremely low trend outlier (−40%, Svratouch, CZ4) due to a near order-of-magnitude decrease in the concentration data series between December 2015 and January 2016, which may have been due to an analytical or reporting error. Overall, these trends correspond to a median 1-Jan-2019 ∑_9_PBDE concentration of 0.30 pg/m^3^ (IQR = 0.12–0.55 pg/m^3^). Schuster et al. observed a steeper decline in atmospheric ∑_6_PBDE concentrations (*t*_1/2_ = 2.2 yr) at 11 sites in the UK and Norway during the period 2000–2008,^43^ which likely captured the initial effects of production and use peaking in the 1990s compared to our later monitoring period of 2011–2019. At Zeppelin (NO2), Wöhrnschimmel et al. reported similar halving times of 1–5 years for BDE 99 and 5–10 years for BDE 47 over the period 2006–2013,^7^ while Wong et al. reported longer halving times of approximately 5 years for BDEs 99, 100, and 138, and 13 years for BDE-47, over a longer monitoring period of 2006–2017.^4^ These results suggest that atmospheric PBDE concentrations at Zeppelin have begun to plateau after an initial decline following the regulation and phasing out of these compounds. This effect is apparent in the lack of significant change and low concentrations observed in our more recent PBDE monitoring data from 2011 to 2019 at the site.

Compared to the other PBDE congeners (∑_9_PBDE), BDE 209 is the most recently regulated POP with long-term monitoring data included in this study, having been listed in the Stockholm Convention in 2017. With use and production not being banned until recently, the trends for BDE 209 vary considerably across the continent and are only statistically significant at four sites. The median annual change is −4.5% (*t*_1/2_ = 15.1 yr), with an IQR of −14.3% to +3.5%, but a total range of −33 to +16%. The absence of a significant trend for BDE 209 is largely consistent with the findings of Wong et al.^4^ Despite the inconsistent trends, the modeled 1-Jan-2019 concentrations are relatively uniform, but approximately 10 times higher than the ∑_9_PBDE concentrations, with a median of 2.0 pg/m^3^ (IQR = 1.1–4.7 pg/m^3^).

### Organochlorine Pesticides

OCPs comprise the majority of the Stockholm Convention POPs, with aldrin, chlordane, DDT, dieldrin, endrin, heptachlor, HCB, and mirex included from the beginning, chlordecone, HCHs, and PeCB listed in 2009, and endosulfan listed in 2011.

A decreasing trend was observed in ∑_6_DDT concentrations at 28 of the 32 sites, with a median annual change of −4.0% (*t*_1/2_ = 17.0 yr). Although only 12 trends are statistically significant, the decreasing trends are highly consistent across most sites, with an IQR of −6.3% to −2.0%. The decreasing trend at Zeppelin (NO2) is much steeper (*t*_1/2_ = 4.6 yr) than the median, but still generally consistent with the trends in the literature for p,p′-DDT (*t*_1/2_ = 1–5.4 yr) and the other DDT substances (*t*_1/2_ = 5–10 yr) at the same site.^4,7^ Despite the relatively uniform rate of decline across the continent, 1-Jan-2019 ∑_6_DDT concentrations varied substantially, spanning nearly 3 orders of magnitude from 0.5 pg/m^3^ (Pallas, FI) to 247 pg/m^3^ (Leova, MD), with a median of 8.7 pg/m^3^ (IQR = 3.6–32.0 pg/m^3^). The highly elevated concentrations at Leova are likely the result of emissions from OCP stockpiles that remain in the city and other regions of Moldova;^44^ highly elevated DDT concentrations have also been measured in the sediments of the Prut River that flows through the city, as well as other rivers across the country.^45^

The steepest and most statistically significant trends observed in this study are for ∑_4_HCH concentrations, with decreases observed at all but one site (Zagreb, HR), with a median annual change of −18.5% (*t*_1/2_ = 3.4 yr) and an IQR of −21.0% to −13.8%, corresponding to a median 1-Jan-2019 ∑_4_HCH concentration of 8.0 pg/m^3^ (IQR = 4.9–12.0 pg/m^3^). At Zeppelin, the ∑_4_HCH trend (*t*_1/2_ = 5.4 yr) was highly consistent with the trends reported by Wong et al. for α-HCH (*t*_1/2_ = 5.3 yr) and γ-HCH (*t*_1/2_ = 4.4 yr) at the same site.^4^ Zagreb is the one major outlier, with a 1-Jan-2019 ∑_4_HCH concentration of 818 pg/m^3^ (the highest of any POP included in this study), which is nearly 20 times higher than the next highest 1-Jan-2019 ∑_4_HCH concentration (51.4 pg/m^3^ at Zmiinyi Island, UA). Concentrations of ∑_4_HCH at Zagreb during the initial years of APOPSBAL/MONET sampling (2004–2007) were significantly lower (88–114 pg/m^3^) and dominated by γ-HCH (55–70%), consistent with the sampling results of Romanić et al. during a similar period in the city (2007–2008).^46^ However, since 2011, the ∑_4_HCH concentrations at the site are highly elevated due to a substantial increase in α-HCH (as high as 3400 pg/m^3^ in 2018), which is now the dominant isomer (∼90%) and indicates that the site has been contaminated by local emissions of α-HCH.

Trends for atmospheric HCB and PeCB concentrations were nearly identical: decreases in HCB were observed at 29 of the 32 sites (23 statistically significant) with a median annual change of −8.5% (*t*_1/2_ = 7.8 yr) and an IQR of −11.0% to −4.0%; decreases in PeCB were observed at 31 of the 32 sites (21 statistically significant) with a median annual change of −9.0% (*t*_1/2_ = 7.3 yr) and an IQR of −11.0% to −5.5%. As with the other POPs, our shallow trend for HCB at Zeppelin (NO2) is consistent with previous findings at the site.^4,7^ Median 1-Jan-2019 concentrations are 34.5 pg/m^3^ (IQR = 25.3–44.3 pg/m^3^) and 9.7 pg/m^3^ (IQR = 6.9–12.2 pg/m^3^) for HCB and PeCB, respectively. These compounds show the narrowest range of concentrations of all POPs (Figure 1), indicating a very uniform distribution across the continent.

### Other POPs

The other POPs included in this study have data series that are too short to determine temporal trends with any certainty. Annual changes in ∑_3_HBCDD concentrations are particularly uncertain—with wide confidence intervals and a lack of statistical significance at most sites—and range from −65% to +77%. However, the modeled 1-Jan-2019 concentrations are very similar to those of the other brominated compounds (∑_9_PBDE) with a median of 0.5 pg/m^3^. Decreasing trends were observed for the cyclodiene OCPs (∑_3_chlordane, dieldrin, ∑_3_endosulfan, and ∑_3_heptachlor) at nearly all sites, with similar median modeled 1-Jan-2019 concentrations of 1.1, 1.8, 0.6, and 1.2 pg/m^3^, respectively. No clear trend was observed for mirex; modeled 1-Jan-2019 concentrations are approximately 1 order of magnitude lower than those of the cyclodiene OCPs, with a median of 0.1 pg/m^3^. Overall, these results suggest that atmospheric concentrations for these OCPs will continue to decrease and remain low over time. Atmospheric concentrations across Europe for the remaining OCPs were consistently below their limits of quantification, which prevented trend analysis for aldrin, chlordecone, and ∑_3_endrin (Table S6). Finally, PFOA and PFOS were monitored for less than 2 years (2013–2015), with median concentrations of 2.6 and 1.1 pg/m^3^, respectively.

### Spatial Analysis

**Figure 2:**
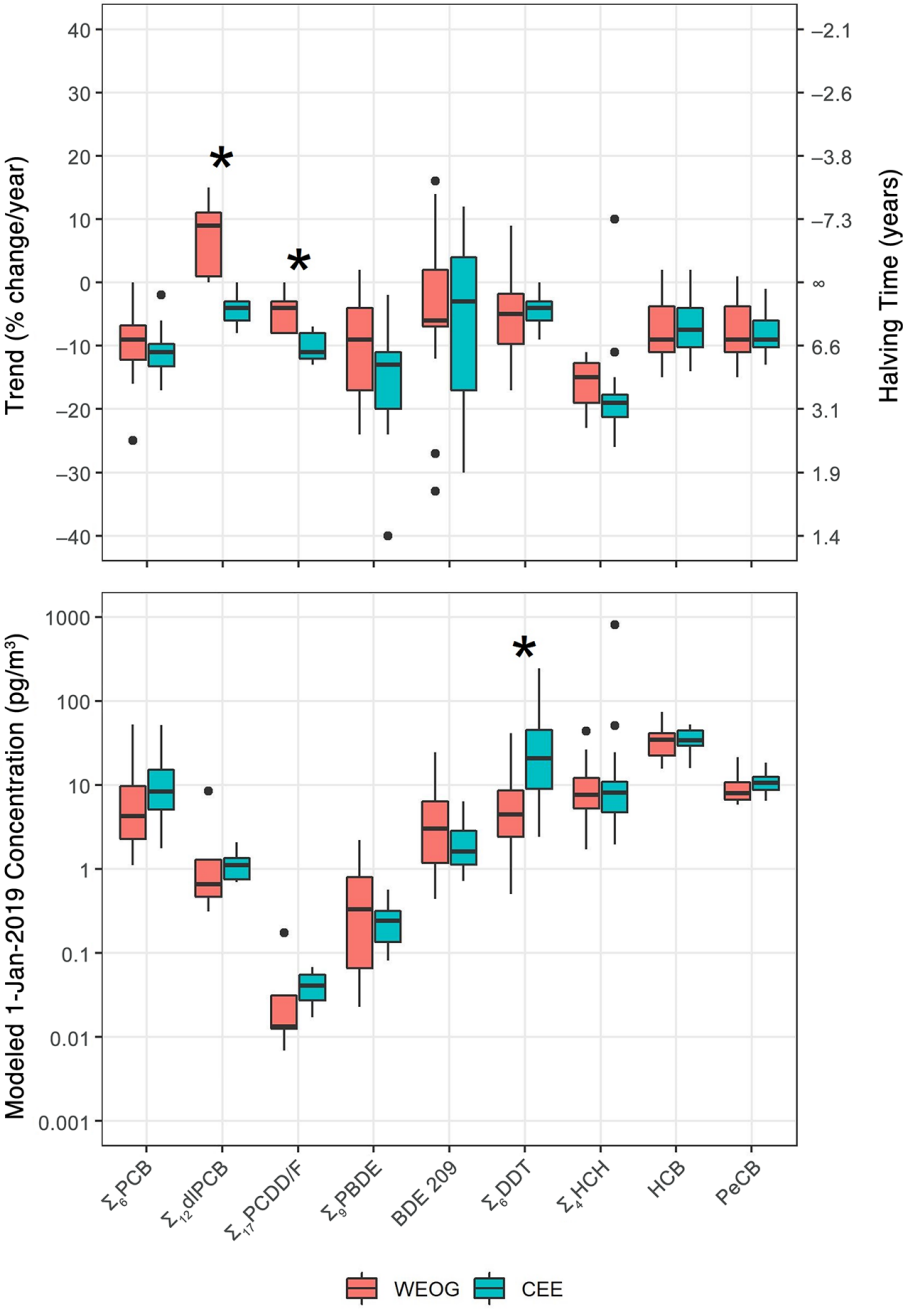
Regional comparison of the temporal trends (% change/year and halving time; Table 2) and the modeled 1-Jan-2019 concentrations (pg/m^3^; Table 3) for POPs at MONET sites in WEOG countries and CEE countries with at least 7 years of continuous atmospheric monitoring data (Table 1). Boxes represent the 25th to 75th percentiles, with the median (50th percentile) represented by a horizontal black line. Whiskers represent the largest/smallest values no further than 1.5 × IQR from the upper/lower limits of the box; values outside of this range are considered outliers and are represented as black dots. * Denotes comparisons that are significantly different (*p* < 0.05).

Despite regional differences in the production, use, and bans of POPs across Europe, no statistically significant differences were observed in the temporal trends or modeled 1-Jan-2019 concentrations between MONET sites in WEOG countries vs. sites in CEE countries for most POPs (Figure 2). The one major exception is that as of January 1, 2019, concentrations of ∑_6_DDT remained significantly elevated at CEE sites (median = 22.3 pg/m^3^, IQR = 9.0–45.3 pg/m^3^) compared to WEOG sites (median = 4.5 pg/m^3^, IQR = 2.4–8.7 pg/m^3^). On the other hand, temporal trends for ∑_6_DDT are consistent between the two regions (median decreases of 4–5% per year), suggesting that the elevated concentrations in CEE are due to the historically higher atmospheric burden in this region.^2^ The only other statistically significant regional difference is that trends for ∑_12_dlPCB and ∑_17_PCDD/F are less negative or even positive at sites in WEOG compared to CEE. However, it is important to reiterate that these compounds were monitored at significantly fewer sites (5 sites in WEOG and 7 sites in CEE) compared to the other POPs (16 sites each in WEOG and CEE for PCBs and OCPs), so that the distributions of the trends shown in Figure 2 may not be as representative of each region compared to the other POPs, making the regional comparison more uncertain.

**Figure 3:**
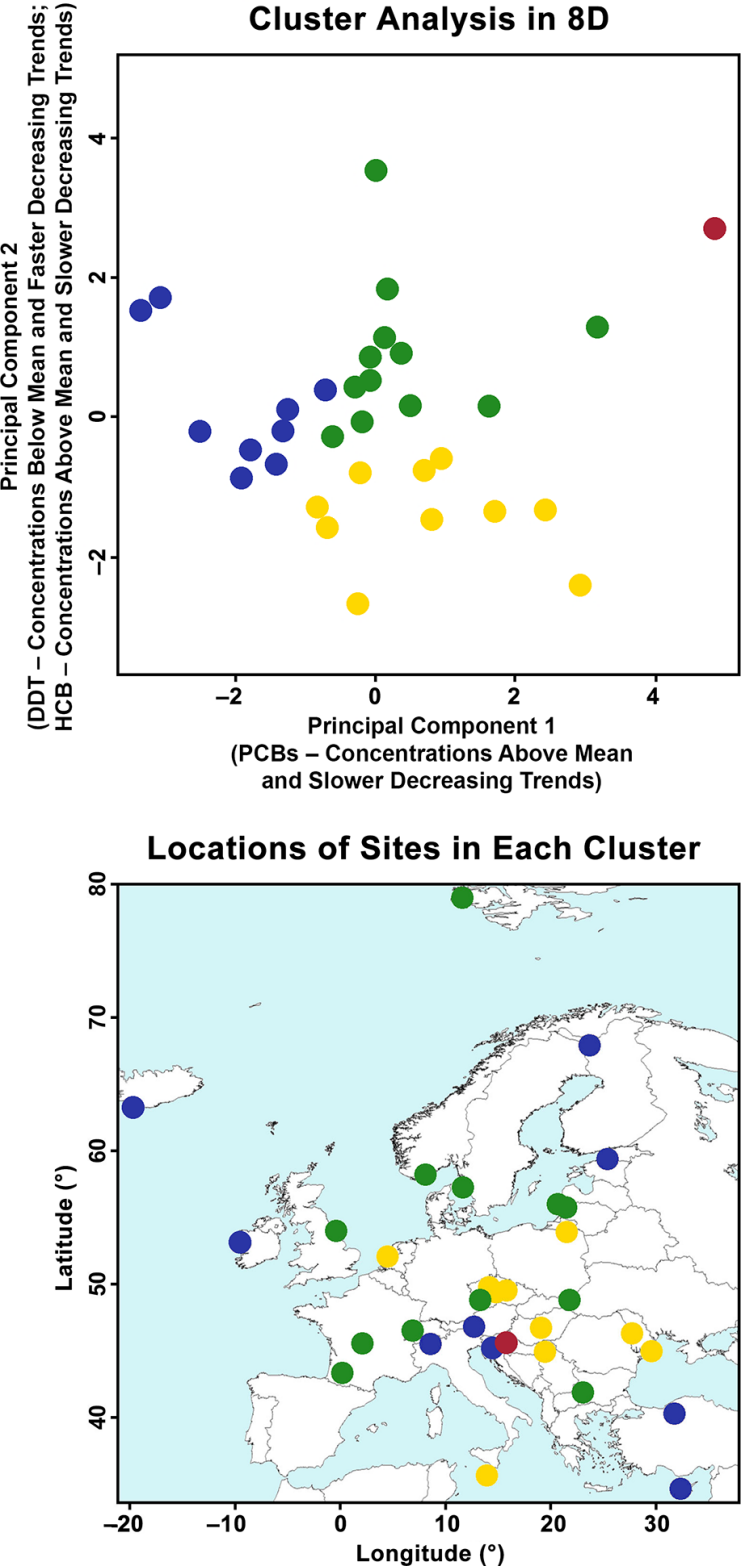
Results of *k*-means clustering with four clusters distinguished by color. The top panel shows the 32 sites as points in 8D space projected to 2D space based on a principal component analysis with the first component (correlated positively with increasing ∑_6_PCB trends) explaining 39% and the second component (correlated positively with 1-Jan-2019 HCB concentrations and increasing trends, and negatively with 1-Jan-2019 ∑_6_DDT concentrations and increasing trends) explaining 24% of the total variability. The bottom panel shows the distribution of the clusters across Europe.

The lack of apparent regional differences in the temporal trends and concentrations of POPs is supported by the results of the continental transect analysis, which were largely inconclusive. Apart from DDT, there are no systematic spatial trends in the location and direction of the transects dividing the continent into halves by maximum differences between concentrations or trends (Figure S4). The cluster analysis, on the other hand, identified four distinct clusters (Figure 3), consistent with some variability in the trends and concentrations, as seen in Figures 1 and 2. Differences between sites (and clusters) may be explained by local site conditions (e.g., elevation, meteorology, land use, emissions, etc.), similar to our findings for POPs on a national scale at MONET sites within the Czech Republic.^16,17^ Input parameters for the cluster analysis, as well as the elevation and wind speed at each site, are listed in Table S7.

The first cluster (blue; 9 sites) is characterized by the lowest POP concentrations and steepest decreasing trends. Sites in this cluster are located across the entire continent in remote or rural areas and have a higher median elevation than the other clusters, consistent with our previous findings in the Czech Republic that more remote MONET sites at higher elevations tend to have lower atmospheric burdens of POPs such as PCBs and DDT.^17^ These sites likely represent continental background conditions and may be more influenced by long-range transport. The second cluster (green; 12 sites) is the largest and is characterized by more moderate concentrations and shallower trends of ∑_6_PCB, ∑_6_DDT, and ∑_4_HCH. Like the blue cluster, sites in the green cluster are located across the entire continent in remote or rural areas, but they tend to be at somewhat lower elevations. The major difference is that this cluster has significantly higher concentrations and shallower trends of HCB compared to the other clusters. The third cluster (red) contains the single urban site included in this study, Zagreb (HR), due to the significantly elevated concentrations of ∑_4_HCH, as discussed previously. The final cluster (yellow, 10 sites) is characterized by the highest concentrations and most shallow trends of ∑_6_PCB, ∑_6_DDT, and ∑_4_HCH; concentrations of ∑_6_DDT are particularly high (median 44.4 pg/m^3^) compared to the blue (median 3.5 pg/m^3^) and green (median 7.5 pg/m^3^) clusters. Apart from De Zilk (NL) and Ġordan Lighthouse (MT), all sites in this cluster are in CEE countries, consistent with the results of the regional comparison and continental transects that DDT is the one POP that remains significantly elevated in this region compared to the rest of the continent.

It is important to note that De Zilk (NL) has the highest concentrations of many of the POPs, including ∑_6_PCB, ∑_12_dl-PCB, ∑_17_PCDD/F, and ∑_9_PBDE and is also one of the windiest sites (median wind speed of 5.3 m/s). Significant variation in wind speed is known to affect the calculation of effective sampling volumes of passive air samplers,^37^ and we have recently found that concentrations of POPs from passive air sampling may be overestimated at coastal MONET sites when wind speeds exceed ∼4 m/s,^27^ consistent with the observations of Tuduri et al.^47^ Median wind speeds at several of the coastal sites in this study exceed this threshold (Mace Head, De Zilk, and Stórhöfdi above 5 m/s; High Muffles, Zmiinyi Island, Lahemaa, and Ġordan Lighthouse above 4 m/s) which suggests that the elevated concentrations at some of these sites may be overestimated and should be interpreted with caution. However, this potential overestimation is consistent across the entire data series for each specific site and compound; thus, the influence on the calculated temporal trends is negligible.^25,27^

### Implications for Future Monitoring

The significant decline in concentrations of nearly all POPs over the past 15 years of MONET monitoring demonstrates that regulatory measures in Europe have been successful in reducing the atmospheric burden of these compounds. Furthermore, despite regional differences in use, production, and elimination, there are no longer any significant differences in the concentrations or trends of POPs between Western, Central, and Eastern European countries (except for DDT). Instead, the extent of heterogeneity observed in the concentrations and trends across the continent for each chemical is driven by local conditions (e.g., elevation, meteorology, land use, emissions, etc.) rather than the geographic location of the sites. Finally, this study highlights the strength of a long-term continental monitoring network where samples are all analyzed using the same methodology by a single laboratory to generate internally consistent data and trends.^48^

We now recommend that the continued long-term monitoring of the legacy POPs for effectiveness evaluation of the Stockholm Convention across Europe is coordinated at the European level, rather than the current patchwork of transient national monitoring programs. Under this proposed framework, monitoring would only be required at a selection of sites of different types (e.g., remote, populated, coastal, mountain, etc.) since similar types of sites located in multiple countries may provide redundant information for the legacy POPs considered here. Importantly, this recommendation only applies to the legacy POPs. For newly listed POPs and other semivolatile organic chemicals of concern (e.g., brominated and organophosphorus flame retardants, volatile PFASs, etc.), country- and regional-specific data will continue to be needed that reflect the spatial pattern of ongoing uses and primary emissions across the continent.

## Supporting Information Available

Additional details on sampling sites, sampling regimes, analytical methods, and trend and spatial analyses (PDF)
Trends and concentrations for individual compounds at each site, as well as more detailed statistical results (XLSX)
