# Supplementary material for: Spatial and Temporal Trends of Persistent Organic Pollutants across Europe after 15 Years of MONET Passive Air Sampling
Source: Environ Sci Technol. 2023 Jul 26;57(31):11583–94. doi: 10.1021/acs.est.3c00796 (PMC10413948; doi:10.1021/acs.est.3c00796)
Supplement: Supplementary file 1 — es3c00796_si_001.pdf [file es3c00796_si_001.pdf]

Spatial and Temporal Trends of Persistent Organic Pollutants across Europe after 15 Years of MONET Passive Air Sampling

Kevin B. White,[†] Jiří Kalina,[†] Martin Scheringer,[*,†,‡] Petra Přibylová,[†] Petr Kukučka,[†] Jiří Kohoutek,[†] Roman Prokeš,[†] and Jana Klánová [*,†]

[†] RECETOX, Masaryk University, Kamenice 753/5, 625 00 Brno, Czech Republic
[‡] Institute of Biogeochemistry and Pollutant Dynamics, ETH Zürich, 8092 Zürich, Switzerland

* Corresponding authors: martin.scheringer@recetox.muni.cz, jana.klanova@recetox.muni.cz

SUPPORTING INFORMATION

TABLE OF CONTENTS

1. MONET S1
Table S1. ..... S1
Table S2 ..... S2
Figure S1 ..... S3
Table S3 ..... S6
2. ANALYTICAL METHODS S7
Table S4 ..... S7
2.1. Chemical Extraction S8
2.2. Instrumental Analysis S9
2.3. QA/QC S12
Table S5 ..... S13
Table S6 ..... S15
3. TREND ANALYSIS S21
Figure S2 ..... S22
Figure S3 ..... S23
4. SPATIAL ANALYSIS S26
4.1. Continental Transects S26
Figure S4 ..... S26
4.2. Cluster Analysis S28
Table S7 ..... S28
6. REFERENCES S29

1. MONET

Table S1. Inactive and short-term monitoring MONET passive air sampling sites in Europe (excluded from this study). Data from most of these sites were published after the initial MONET campaign in Central and Eastern Europe from 2006–2008.[1]

Country	Name	Lat.	Long.	Elevation	Type	Start	End
Belarus	Berezinsky	55.483	28.350	185	Remote	2008	2008
Belarus	Vysokoe	52.333	23.333	151	-	2008	2008
Bulgaria	Pernik	42.588	23.116	760	Urban	2007	2007
Bulgaria	Plovdiv	42.024	24.837	290	Urban	2007	2007
Bulgaria	Sofia, Bojana	42.643	23.264	660	Suburban	2007	2007
Bulgaria	Sofia, Hipodruma	42.511	23.305	545	Urban	2007	2007
France	La Coulonche	48.645	-0.458	309	Rural	2009	2013
Germany	Westerland	54.892	8.325	5	Rural	2009	2012
Greece	Aliartos	38.375	23.110	110	Rural	2009	2010
Hungary	Budapest, Gilice ter	47.430	19.181	134	Suburban	2007	2007
Hungary	Budapest, Kozseghaz u.	47.562	18.961	254	Urban	2007	2007
Italy	Monterotondo	42.106	12.640	200	-	2009	2011
Latvia	Olaine	56.898	23.743	3	Urban	2006	2006
Latvia	Zoseni	57.135	25.906	185	Rural	2006	2006
Lithuania	Paneriai	54.658	25.238	113	Urban	2006	2006
Lithuania	Preila	55.350	21.067	5	Rural	2006	2013
Lithuania	Rugsteliskes	55.441	26.067	194	Rural	2006	2014
Moldova	Stefan Voda	46.500	29.500	169	Rural	2007	2007
Montenegro	Podgorica, CETI	42.425	19.267	54	Suburban	2007	2007
North Macedonia	Bujkovci	42.006	21.653	312	Urban	2007	2007
North Macedonia	Lazaropole	41.537	20.696	1333	Rural	2007	2010
North Macedonia	Strumica	41.442	22.665	232	Rural	2007	2007
Norway	Karvatn	62.783	8.883	210	Remote	2009	2015
Poland	Szarow	49.995	20.267	207	Rural	2007	2007
Poland	Zabierzow	50.109	19.825	221	Rural	2007	2007
Romania	Ruginesti	46.983	26.083	542	Remote	2006	2006
Russia	Chapaevsk	52.952	49.802	115	Urban	2007	2007
Russia	Groznyj	43.332	45.700	117	Urban	2008	2008
Russia	Obninsk	55.100	36.600	176	Urban	2009	2011
Russia	Ufa	54.700	55.800	133	Urban	2007	2007
Slovakia	Bratislava, Mamateyova	48.125	17.126	136	Urban	2006	2006
Slovakia	Handlova, Morovianska	48.733	18.756	440	Urban	2006	2006
Slovakia	Kosice, Strojarska	48.727	21.252	208	Urban	2006	2006
Slovakia	Ruzomberok, Riadok	49.079	19.303	481	Urban	2006	2006
Slovakia	Strazske, Mierova	48.874	21.837	133	Urban	2006	2006
Slovakia	Topolniky	47.959	17.860	112	Rural	2006	2006

Slovakia	Ziar nad Hronom, Dukelskych hrdinov	48.584	18.851	243	Suburban	2006	2006
Slovakia	Zilina, Obezna	49.211	18.771	357	Urban	2006	2006
Slovenia	Celje, east	46.242	15.295	239	Urban	2007	2007
Slovenia	Celje, south	46.234	15.262	212	Urban	2007	2007
Slovenia	Ljubljana	46.066	14.513	297	Urban	2007	2007
Slovenia	Maribor	46.541	15.684	255	Urban	2007	2007
Spain	Donana National Park	36.988	-6.405	-	-	2009	2010
Spain	Izana Station	28.309	-16.499	2400	-	2010	2011
Sweden	Aspvreten	58.806	17.388	25	Remote	2009	2013
Ukraine	Lisove	50.467	30.111	149	Suburban	2008	2008
Ukraine	Oseshchyna	50.581	30.532	94	Suburban	2008	2008
Ukraine	Petrodolinskoe	46.456	30.336	66	Rural	2009	2014

Table S2. MONET PUF-PAS sampler design.

**PUF disk characteristics**	
Volume (m^3)	2.65×10^{-4}
Diameter (m)	1.50×10^{-1}
Thickness (m)	1.50×10^{-2}
Effective film thickness (m)	5.67×10^{-3}
Density (g/m^3)	3.03×10^{4}
Mass (g)	7.92
**Sampler housing dimensions (cm)**	
Diameter (upper dome)	28
Depth (upper dome)	8
Diameter (lower dome)	22
Depth (lower dome)	8

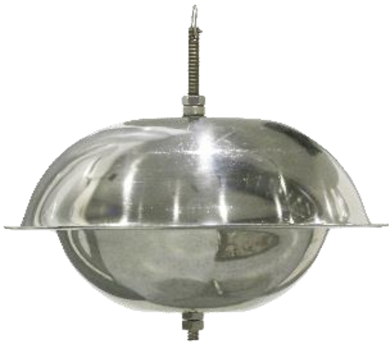

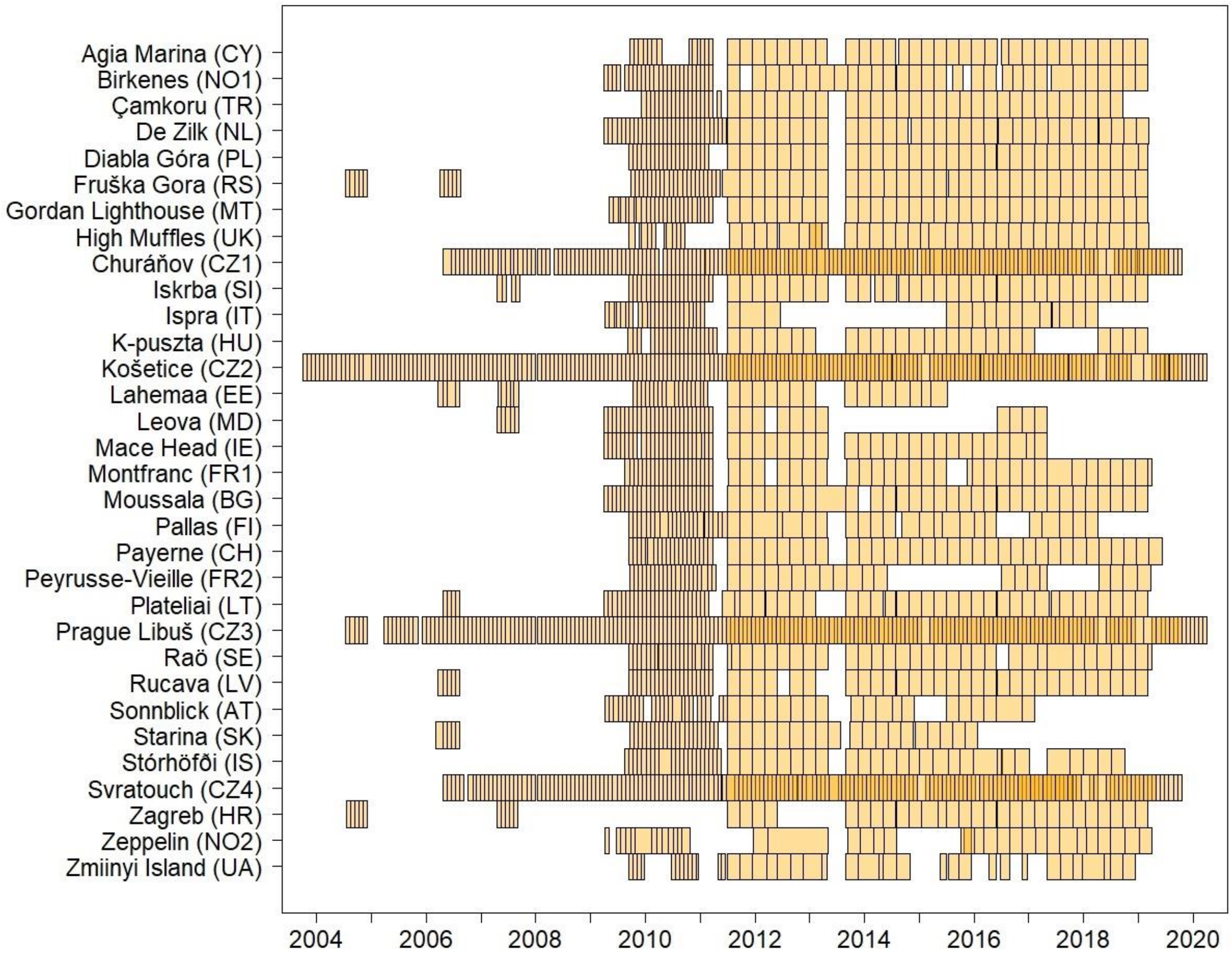

Figure S1A. MONET sampling regime for polychlorinated biphenyls (PCBs) and basic organochlorine pesticides (DDT, HCH, HCB, PeCB)

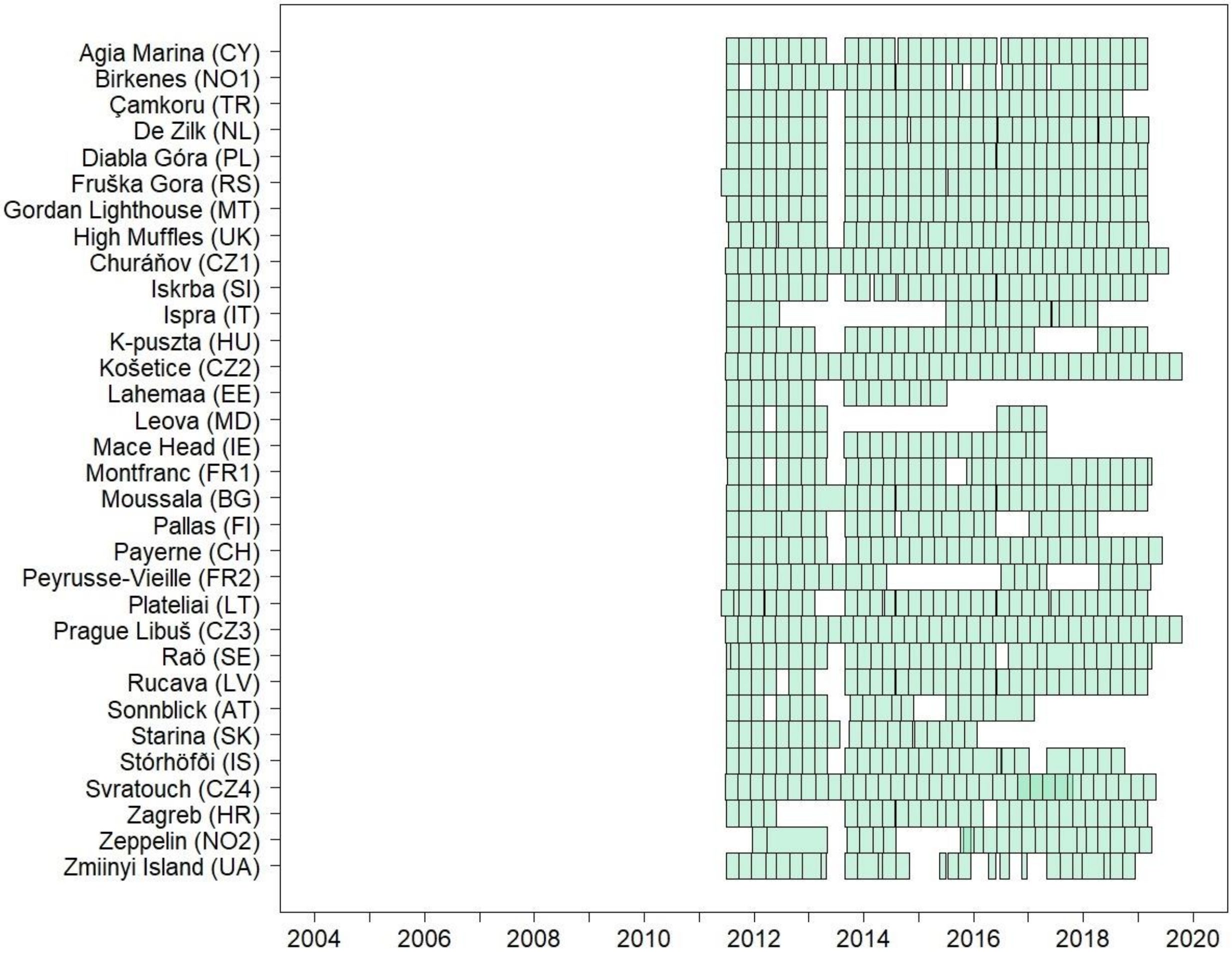

Figure S1B. MONET sampling regime for polybrominated diphenyl ethers (PBDEs).

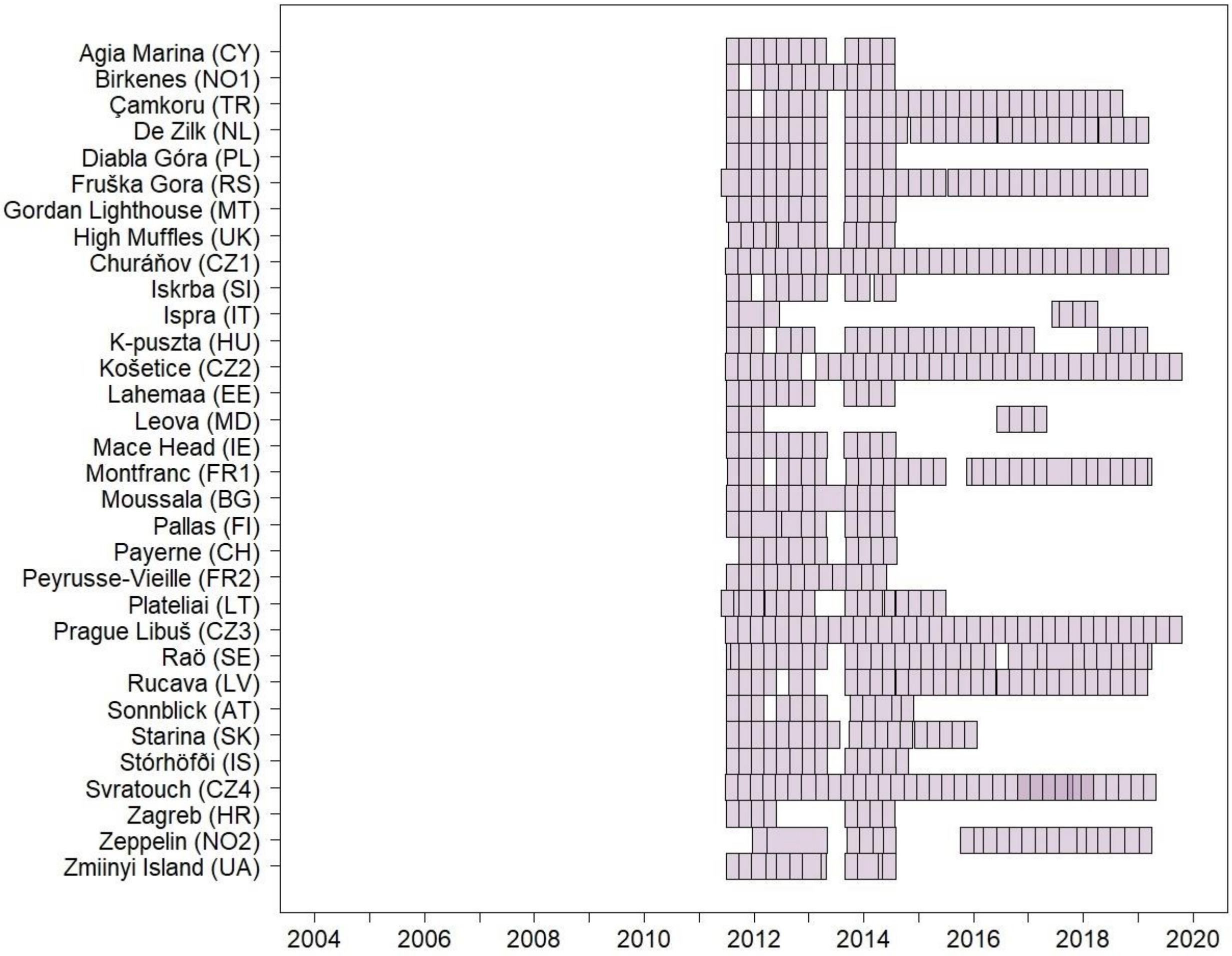

Figure S1C. MONET sampling regime for polychlorinated dibenzo-dioxins/furans (PCDD/Fs) and dioxin-like PCBs (dl-PCBs).

Table S3. Monitoring of the Stockholm Convention Persistent Organic Pollutants in air under the MONET network in Europe

Stockholm Convention POPs	MONET sampling in Europe
**Included in UNEP First Global Monitoring Report (2009)[2] [Entry-into-force May 2004]**	
Aldrin	Since 2011
Chlordane	Since 2011
Dichlorodiphenyltrichloroethane (DDT)	Since 2003
Dieldrin	Since 2011
Endrin	Since 2011
Heptachlor	Since 2011
Hexachlorobenzene (HCB)	Since 2003
Mirex	Since 2011
Polychlorinated biphenyls (PCBs)	Since 2003
Polychlorinated dibenzo dioxins/furans (PCDD/Fs)	Since 2011
Toxaphene	*Not monitored*
**Included in UNEP Second Global Monitoring Report (2017)[3] [Listed May 2009/2011/2013]**	
Chlordecone	2011–2014
Endosulfan	Since 2011
Hexabromobiphenyl	*Not monitored*
Hexabromocyclododecane (HBCDD)	Since 2012
Hexachlorocyclohexanes (HCHs)	Since 2003
Pentachlorobenzene (PeCB)	Since 2003
Perfluorooctanesulfonic acid (PFOS)	2013–2015
Polybrominated diphenyl ethers (PBDEs)	Since 2011
**‘New’ Persistent Organic Pollutants [Listed May 2015/2017/2019/2022]**	
Decabromodiphenyl ether (BDE 209)	Since 2011
Dicofol	*Not monitored*
Hexachloro-1,3-butadiene (HCBD)	*Not monitored*
Pentachlorophenol (PCP)	*Not monitored*
Perfluorooctanoic acid (PFOA)	2013–2015
Perfluorohexane sulfonic acid (PFHxS)	*Not monitored*
Polychlorinated naphthalenes (PCNs)	*Not monitored*
Short-chain chlorinated paraffins (SCCPs)	*Not monitored*

2. ANALYTICAL METHODS

MONET passive air samplers consist of polyurethane foam disks (Table S2; Gumotex, Breclav, Czech Republic) housed in a protective chamber. The sampling chambers were prewashed and solvent-rinsed with acetone prior to installation. All PUF disks were prewashed, cleaned (8 h extraction in acetone, followed by 8 h in dichloromethane, toluene or methanol; depending on the analytes), wrapped in two layers of aluminum foil, placed in zip-lock polyethylene bags and kept in a freezer prior to their deployment. Once installed at the sampling site, PUF disks were exposed for approximately 12 weeks (84 d). Field blanks were obtained by installing and then removing an additional PUF disk at all sampling sites. After sampling, the exposed PUF disks were collected and again wrapped in two layers of aluminum foil, labeled, placed in zip-lock polyethylene bags, and transported in a cooler containing dry ice and a temperature logger to RECETOX where they were kept in a freezer at –18 °C until analysis (shipment generally within 48 hours of collection; samples maintained at 5 °C in transit when possible). All PUF disks were analyzed at the RECETOX Trace Analytical Laboratory in Brno, Czech Republic. Samples were analyzed for the individual compounds and analytes outlined in Tables S3 and S5. The analytical methods described here are the current methods used for analysis of passive PUF samples collected under the MONET network since 2014 (an overview of methods is presented in Table S4). For analytical methods used in the initial MONET campaigns in Central and Eastern Europe, refer to Přibylová et al.[1] One notable change occurred, with the transition from GC-EI-MS/MS to GC-APCI-MS/MS for the analysis of the 'Other OCPs' (Section 2.2.1d.), leading to significantly improved instrumental detection limits after 2013 (Table S6).

Table S4. Overview of analytical methods and instrumentation. Details in Sections 2.1–2.3.

	Standard spike (pre-extraction)	Soxhlet extraction	Standard spike (pre-analysis)	Analysis
PCBs, OCPs	PCB 30, PCB 185	DCM	PCB 121	GC-MS/MS
Other OCPs	PCB 30, PCB 185	DCM	PCB 121	GC-APCI-MS/MS
PBDEs	^{13}C-PBDEs (8 congeners, no BDE 66 or 85)	DCM	^{13}C-BDE 77 and ^{13}C-BDE 138	GC-HRMS
HBCDD	^{13}C-γ-HBCDD	DCM	*None*	HPLC-MS
PCDD/Fs, dl-PCBs	^{13}C-PCDD/Fs (17 homologues), ^{13}C-dl-PCBs (12 congeners)	Toluene	^{13}C-1,2,3,4-TCDD and ^{13}C-PCB 162	2 x GC-HRMS
PFOA, PFOS	^{13}C-8PFOA, ^{13}C-8PFOS	Methanol	*None*	UHPLC-MS

2.1. Chemical Extraction

All samples were spiked with surrogate internal standards before extraction: ^{13}C-labelled BDE 28, 47, 99, 100, 153, 154, 183 and 209 congeners; ^{13}C-HxBB-153, ^{13}C-γ-HBCDD, ^{13}C-dl-PCBs (77, 81, 105, 114, 118, 123, 126, 156, 157, 167, 169, and 189), ^{13}C-PCDD/F homologues, ^{13}C-8PFOA, and ^{13}C-8PFOS. All ^{13}C-labelled standards were obtained from Wellington Laboratories Inc., Canada. For PCB and OCP analysis, the surrogate standards, PCB 30 and 185, were used (Absolute Standards Inc., USA). Three subsamples of each PUF disk were Soxhlet-extracted (Büchi B-811, Switzerland) in dichloromethane (DCM), toluene, and methanol, respectively. DCM extracts were split into two aliquots: 60% for the analysis of indicator PCBs (in-PCBs), OCPs (DDT, HCB, HCH, PeCB), PBDEs, and HBCDD; 40% for the analysis of the ‘other’ OCPs (aldrin, chlordane, chlordecone, dieldrin, endosulfan, endrin, heptachlor, and mirex). Toluene extracts were used for the analysis of PCDD/Fs and dioxin-like PCBs (dl-PCBs). Methanol extracts were used for the analysis of PFOS and PFOA.

2.1.1. Clean-up of DCM extracts (in-PCBs, OCPs, PBDEs, and HBCDD)

Each aliquot for in-PCB, OCP, PBDE, HxBB-153, and HBCDD analysis (60% of the original DCM extract) was cleaned on sulfuric acid modified silica columns eluted with 40 mL of a 1:1 mixture of *n*-hexane:DCM.[4] The eluate volume was reduced to 1 mL by a stream of nitrogen gas in a TurboVap II (Caliper LifeSciences, USA) concentrator unit and transferred into a vial. All eluate volumes (except those for HBCDD analysis via HPLC) were further reduced to 150 µL in a LabEva concentrator (HPST, Czech Republic). PCB 121 was then added as a syringe (recovery) standard prior to instrumental analysis.

Each aliquot for ‘other’ OCP analysis (40% of DCM extract) was cleaned on a silica column (5 g of silica, 0.063 – 0.200 mm, activated at 150°C for 12 hours, then 10% deactivated with water) and 1 g Na_2SO_4. The sample was loaded and eluted with 5 mL *n*-hexane followed by 50 mL DCM. The eluate volume was reduced to 1 mL by a stream of nitrogen gas in a TurboVap II (Caliper LifeSciences, USA) concentrator unit and transferred into a vial where it was reduced further to 150 µL in a LabEva concentrator (HPST, Czech Republic). PCB 121 was then added as a syringe (recovery) standard prior to instrumental analysis.

2.1.2. Clean-up and fractionation of toluene extracts (PCDD/Fs and dl-PCBs)

Each toluene extract for PCDD/F and dl-PCB analysis was cleaned on a sulfuric acid modified silica column (30% w/w), eluted with a 40 mL DCM/*n*-hexane mixture (1:1). Fractionation was achieved in a micro column (6 mm i.d) containing from bottom to top: 50 mg silica, 70 mg charcoal/silica (1:40), and 50 mg of silica gel. The column was prewashed with 5 mL of toluene, followed by 5 mL of a DCM/cyclohexane mixture (30%), then the sample was added and eluted with 9 mL of a DCM/cyclohexane mixture (30%) in Fraction #1 (mono-ortho dl-PCBs) and 40 mL of toluene in Fraction #2 (PCDD/Fs, non-ortho dl-PCBs). Each fraction was concentrated to 1 mL using a stream of nitrogen gas in a TurboVap II (Caliper LifeSciences, USA) concentrator unit and transferred into a vial where it was reduced further to 10 µL in a LabEva concentrator (HPST, Czech Republic). ^{13}C-1,2,3,4-TCDD and ^{13}C-PCB 162 were then added as syringe (recovery) standards prior to instrumental analysis.

2.1.3. Clean-up of methanol extracts (PFOA and PFOS)

Each methanol extract for PFOA and PFOS analysis was cleaned using a syringe filter (nylon membrane, 13 mm diameter, 0.45 µm pore size) and transferred into a polypropylene centrifuge tube. Filtrates in centrifuge tubes were concentrated to 1 mL under a gentle stream of nitrogen gas. Samples were diluted using a solution of ammonium acetate ($NH_4CH_3CO_2$) in water (5 mM) up to final volume of 50/50 (v/v) $NH_4CH_3CO_2$ in water/$NH_4CH_3CO_2$ in methanol. Samples were centrifuged for 10 min at 4000 rpm, then 100 µL aliquots were transferred to vials for analysis.

2.2. Instrumental Analysis

2.2.1a. Indicator PCBs and OCPs (DDT, HCB, HCH, PeCB)

Each DCM extract for in-PCBs, OCPs, PBDEs, HxBB-153, and HBCD was first analyzed for in-PCBs and OCPs by gas chromatography tandem mass spectrometry (GC-MS/MS) using an Agilent 7890A GC (Agilent, USA) equipped with a 60 m x 0.25 mm x 0.25 µm SGE-HT8 column (SGE, USA), coupled to an Agilent 7000B triple quadrupole MS operating in positive electron ionization (EI+) multiple reaction monitoring (MRM) mode. Samples were splitless injected at 280°C, with He as the carrier gas at a constant flow of 1.5 mL/min. The GC temperature program was 80 °C (1 min hold), ramp 15 °C/min to 180°C, and final ramp 5 °C/min to 300°C (5 min hold).

2.2.1b. PBDEs

Extracts were then analyzed for PBDEs by gas chromatography high-resolution mass spectrometry (GC-HRMS) using an Agilent 7890A GC equipped with a 15 m x 0.25 mm x 0.10 µm RTX-1614 column (Restek, USA), coupled to a Waters AutoSpec Premier MS (Waters, UK) operating in EI+ selected ion monitoring (SIM) mode at a resolution of >10,000 and 35 eV electron energy; for BDE 209, the resolution was set to >5,000. Samples were splitless injected at 280 °C, with He as the carrier gas at a constant flow of 1 mL/min, and increased to 1.4 mL/min after 15 min. The GC temperature program was 80 °C (1 min hold), ramp 20 °C/min to 250 °C, ramp 1.5 °C/min to 260 °C (2 min hold), and final ramp 25 °C/min to 320°C (4.5 min hold). Waters TargetLynx was used for data processing.

After analysis of the 2.2.1a and 2.2.1b compounds, the remaining extract was solvent exchanged to acetonitrile, and the syringe standard ^{13}C-γ-HBCDD was added before final HBCDD analysis.

2.2.1c. HBCDD

Extracts were analyzed for HBCDD by high performance liquid chromatography mass spectrometry (HPLC-MS) using an Agilent 1100 HPLC equipped with a Phenomenex LUNA C18 end-capped column (100 × 2 mm, 3 µm particle size) maintained at 30 °C and a Phenomenex SecureGuard C18 pre-column, coupled to a tandem AB Sciex QTRAP 5500 MS (AB Sciex, Canada) operating in EI− MRM mode at 450°C, with N_2 as a nebulizer gas and an entrance potential of -4kV. The mobile phases for the gradient separation were a 1 mM solution of ammonium acetate in water at pH 4 (solution A) and a 1 mM solution of ammonium acetate in acetonitrile (solution B). The flow rate was 0.25 mL/min, and the injection volume was 10 µL. The linear gradient began at an initial A/B composition of 50:50 (v/v) and ran to 10:90 over 6 min, where it was held for 8 min.

2.2.1d. Other OCPs (aldrin, chlordane, chlordecone, dieldrin, endosulfan, endrin, heptachlor, mirex)

Each DCM extract for other OCPs was analysed by gas chromatography atmospheric pressure chemical ionization tandem mass spectrometry (GC-APCI-MS/MS) using an Agilent 7860A GC equipped with a 30 m x 0.25 mm x 0.25 µm Rxi-5Sil MS column (Restek, USA), coupled to a Waters Xevo TQ-S MS operating under dry source conditions (N_2 at 40 psi constant pressure) in MRM mode. Samples were splitless injected at 250°C, with He as the carrier gas at a constant flow of 1.5 mL/min. The GC temperature program was 90 °C (1 min hold), ramp 40 °C/min to 200 °C, ramp 2 °C/min to 240 °C, and final ramp 40 °C/min to 310 °C (5 min hold).

2.2.2. PCDD/Fs and dioxin-like PCBs (dl-PCBs)

Each toluene extract for PCDD/Fs and dl-PCBs was analyzed by 2xGC-HRMS using dual Thermo Scientific TRACE 1310 GCs (Thermo Fisher Scientific, USA) equipped with a 60 m × 0.25 mm × 0.25 µm Restek Rtx-Dioxin2 column (for PCDD/Fs) and a 60 m × 0.25 mm × 0.25 µm SGE HT8 column (for dl-PCBs), coupled to a Thermo Scientific DFS HRMS operating at a resolution of 10,000 (10% valley) and 45 eV electron energy. PCDD/F samples were splitless injected at 280 °C and dl-PCB samples were splitless injected at 260 °C, with He used as the carrier gas for both at a constant flow of 1.5 mL/min. The GC temperature program for PCDD/Fs was 120 °C (1.5 min hold), ramp 30 °C/min to 200 °C, ramp 3 °C/min to 300 °C (2.5 min hold), and final ramp 30 °C/min to 320 °C (20 min hold). The GC temperature program for dl-PCBs was 120 °C (1.5 min hold), ramp 30 °C/min to 200 °C, and final ramp 3 °C/min to 310 °C (10 min hold). Thermo Scientific TargetQuan 3 was used for data processing.

2.2.3. PFOA and PFOS

Each methanol extract for PFOA and PFOS was analyzed by ultra high-performance liquid chromatography mass spectrometry (UHPLC-MS) using an Agilent 1290 UHPLC equipped with a SYNERGI 4µ Fusion RP 80Ä 50 mm x 2 mm column and corresponding pre-column 4 mm x 2.00 mm (Phenomenex, USA), coupled with a SCIEX 5500 QTRAP MS (SCIEX, Canada) operating in ESI− mode using two MRM transitions at 450°C and ion voltage 4500 V. The flow rate was set to 200 µL/min. The mobile phase A was methanol/5 mM ammonium acetate in water 55/45 (% v/v), and the mobile phase B was methanol. Aliquots of 10 µL were injected onto the column and directly after injection the linear gradient was raised from 0% A to 70% A within 4 min and was held for 2 min. Then the percentage of A was linearly decreased to 0% and was held for 1.5 min. Before the next separation, the column was equilibrated using the initial content of the mobile phase for 3.5 min.

2.3. QA/QC

Unexposed PUF disks transported and processed alongside the other samples were used as field blanks. The concentrations in MONET field blanks were <5% for PBDEs (<20% for BDE 209), and below detection for all PCBs, OCPs, and PCDD/Fs. Field blanks were used to determine method detection limits (MDLs), based on the average + 3x the standard deviation of the field blanks. For compounds that were not detected in the field blanks, the instrument quantitation limits (LOQs) were determined as concentrations corresponding to a 9:1 signal to noise ratio. Laboratory method blanks were also analyzed with each sample set (one laboratory blank per 20 samples).

Method performance was tested prior to sample preparation and analysis using reference materials. Recoveries of in-PCBs and OCPs were monitored using PCB 30 and 185 recovery standards, with an average PCB 30 recovery of 66.2 ± 25.9% and an average PCB 185 recovery of 98.0 ± 17.6%. PCB and OCP concentrations were not further adjusted for recoveries. Isotope dilution was used to quantify PBDEs, HBCDD, PCDD/Fs, dl-PCBs, PFOS and PFOA, thus the reported concentrations were inherently adjusted for recoveries.

Table S5. POP analytes in air and their corresponding abbreviations. Trends and concentrations of the ‘Other OCPs” were significantly affected by poor instrumental detection for samples collected and analyzed prior to 2014 (see Table S6).

Class	Compound	Abbreviation
Indicator PCBs $\sum_6$PCB	2,4,4'-trichlorobiphenyl	PCB 28
	2,2',5,5'-tetrachlorobiphenyl	PCB 52
	2,2',4,5,5'-pentachlorobiphenyl	PCB 101
	2,2',3,4,4',5'-hexachlorobiphenyl	PCB 138
	2,2',4,4',5,5'-hexachlorobiphenyl	PCB 153
	2,2',3,4,4',5,5'-heptachlorobiphenyl	PCB 180
OCPs $\sum_4$HCH $\sum_6$DDT	pentachlorobenzene	PeCB
	hexachlorobenzene	HCB
	α-hexachlorocyclohexane	α-HCH
	β-hexachlorocyclohexane	β-HCH
	γ-hexachlorocyclohexane	γ-HCH
	δ-hexachlorocyclohexane	δ-HCH
	o,p'-dichlorodiphenyldichloroethane	o,p'-DDD
	p,p'-dichlorodiphenyldichloroethane	p,p'-DDD
	o,p'-dichlorodiphenyldichloroethylene	o,p'-DDE
	p,p'-dichlorodiphenyldichloroethylene	p,p'-DDE
	o,p'-dichlorodiphenyltrichloroethane	o,p'-DDT
	p,p'-dichlorodiphenyltrichloroethane	p,p'-DDT
Other OCPs $\sum_3$Chlordane $\sum_3$Endosulfan $\sum_3$Endrin $\sum_3$Heptachlor	aldrin	ALD
	α-chlordane	A_CHL
	γ-chlordane	G_CHL
	oxychlordane	OXCHL
	chlordecone	CHLODE
	dieldrin	DIE
	α-endosulfan	A_ENDS
	β-endosulfan	B_ENDS
	endosulfan sulfate	ENDS_SUL
	endrin	END
	endrin aldehyde	END_AL
	endrin ketone	END_KE
	heptachlor	HEP
	cis-heptachlor epoxide	CIS_HEP
	trans-heptachlor epoxide	TRANS_HEP
	mirex	MIREX
PBDEs $\sum_9$PBDE (excluding 209)	2,4,4’-tribromodiphenyl ether	BDE 28
	2,2',4,4'-tetrabromodiphenyl ether	BDE 47
	2,3’,4,4’-tetrabromodiphenyl ether	BDE 66
	2,2’,3,4,4’-pentabromodiphenyl ether	BDE 85
	2,2’,4,4’,5-pentabromodiphenyl ether	BDE 99
	2,2’,4,4’,6-pentabromodiphenyl ether	BDE 100
	2,2’,4,4’,5,5’-hexabromodiphenyl ether	BDE 153
	2,2’,4,4’,5,6’-hexabromodiphenyl ether	BDE 154
	2,2′,3,4,4′,5′,6-heptabromodiphenyl ether	BDE 183
	decabromodiphenyl ether	BDE 209

HBCDD<br>$\sum_3$HBCDD	α-hexabromocyclododecane	α-HBCDD
	β-hexabromocyclododecane	β-HBCDD
	γ-hexabromocyclododecane	γ-HBCDD
PCDD/Fs<br>$\sum_7$PCDD<br>$\sum_{10}$PCDF<br>$\sum_{17}$PCDD/F	2,3,7,8-tetrachlorodibenzo-p-dioxin	2378-TCDD
	1,2,3,7,8-Pentachlorodibenzo-p-dioxin	12378-PeCDD
	1,2,3,4,7,8-Hexachlorodibenzo-p-dioxin	123478-HxCDD
	1,2,3,6,7,8-Hexachlorodibenzo-p-dioxin	123678-HxCDD
	1,2,3,7,8,9-Hexachlorodibenzo-p-dioxin	123789-HxCDD
	1,2,3,4,6,7,8-Heptachlorodibenzo-p-dioxin	1234678-HpCDD
	octachlorodibenzo-p-dioxin	OCDD
	2,3,7,8-tetrachlorodibenzofuran	2378-TCDF
	1,2,3,7,8-pentachlorodibenzofuran	12378-PeCDF
	2,3,4,7,8-pentachlorodibenzofuran	23478-PeCDF
	1,2,3,4,7,8-hexachlorodibenzofuran	123478-HxCDF
	1,2,3,6,7,8-hexachlorodibenzofuran	123678-HxCDF
	2,3,4,6,7,8-hexachlorodibenzofuran	234678-HxCDF
	1,2,3,7,8,9-hexachlorodibenzofuran	123789-HxCDF
	1,2,3,4,6,7,8-heptachlorodibenzofuran	1234678-HpCDF
	1,2,3,4,7,8,9-heptachlorodibenzofuran	1234789-HpCDF
	octachlorodibenzofuran	OCDF
Dioxin-like PCBs<br>$\sum_{12}$dl-PCB	3,3',4,4'-tetrachlorobiphenyl	PCB 77
	3,4,4',5-tetrachlorobiphenyl	PCB 81
	2,3,3',4,4'-pentachlorobiphenyl	PCB 105
	2,3,4,4',5-pentachlorobiphenyl	PCB 114
	2,3',4,4',5-pentachlorobiphenyl	PCB 118
	2,3',4,4',5'-pentachlorobiphenyl	PCB 123
	3,3',4,4',5-pentachlorobiphenyl	PCB 126
	2,3,3',4,4',5-hexachlorobiphenyl	PCB 156
	2,3,3',4,4',5'-hexachlorobiphenyl	PCB 157
	2,3',4,4',5,5'-hexachlorobiphenyl	PCB 167
	3,3',4,4',5,5'-hexachlorobiphenyl	PCB 169
	2,3,3',4,4',5,5'-heptachlorobiphenyl	PCB 189
PFASs	perfluorooctane sulfonate	PFOS
	perfluorooctanoic acid	PFOA

Table S6A. Percentage (%) of samples at each WEOG MONET site in Europe that fell below the limit of quantification (LOQ) over the entire monitoring period. Site/compounds with more than 30% of samples <LOQ could not be analyzed for temporal trends and are highlighted in yellow. Values in orange depict OCP compounds with poor detection prior to changes in analytical instrumentation.

Class	Compounds	Percentage of samples with concentrations <LOQ (%)															
		AT	CY	FI	FR1	FR2	IS	IE	IT	MT	NL	NO1	NO2	SE	CH	TR	UK
Indicator PCBs	PCB 28	0	0	0	0	0	0	0	0	0	0	3	7	0	0	0	0
	PCB 52	0	0	0	0	0	0	0	0	0	0	5	0	0	0	0	0
	PCB 101	4	5	8	0	0	0	0	0	0	0	12	11	0	0	3	3
	PCB 138	21	14	57	15	16	0	26	0	0	0	24	36	0	13	42	15
	PCB 153	11	5	38	3	0	0	0	0	0	0	12	14	0	3	38	9
	PCB 180	14	34	61	30	10	7	8	0	0	0	41	39	0	23	63	55
OCPs	PeCB	0	0	0	0	0	0	0	0	8	0	0	0	0	0	0	0
	HCB	0	0	0	0	0	0	0	0	0	0	0	0	0	0	0	0
	α-HCH	0	0	0	0	0	0	0	0	0	0	0	0	0	0	0	0
	β-HCH	16	5	17	12	13	21	0	0	0	3	74	58	18	38	3	52
	γ-HCH	0	0	3	0	0	0	0	0	0	0	0	3	0	0	0	0
	δ-HCH	5	39	48	3	0	52	8	27	12	0	68	67	55	46	53	55
	o,p'-DDD	36	20	61	64	41	13	16	0	0	0	20	42	22	43	47	23
	p,p'-DDD	32	19	65	56	17	19	48	0	3	2	35	50	22	28	46	24
	o,p'-DDE	18	0	54	30	17	9	17	5	0	0	27	19	0	43	21	27
	p,p'-DDE	0	0	0	0	0	0	0	0	0	0	0	0	0	0	0	0
	o,p'-DDT	20	0	39	3	0	15	0	0	0	0	9	39	3	0	0	7
	p,p'-DDT	17	0	44	9	9	10	17	0	0	0	14	31	0	8	9	12
Other OCPs [before 2014]	ALD	100	100	100	100	100	100	100	100	100	100	100	100	100	100	100	100
	α-CHL	38	44	100	100	100	0	0	67	0	0	100	50	82	100	100	93
	γ-CHL	63	56	100	100	100	7	0	33	8	0	100	83	94	100	100	93
	OXCHL	100	100	100	100	100	100	100	100	100	100	100	100	100	100	100	100
	DIE	75	94	100	91	58	0	0	100	0	0	100	100	77	83	100	0
	α-ENDS	0	13	86	64	50	0	0	0	0	0	100	83	77	33	36	87
	β-ENDS	88	88	100	100	100	100	100	100	83	100	100	100	100	100	100	100
	ENDS_SUL	88	100	100	100	100	100	100	100	83	100	100	100	100	100	100	100
	END	100	100	100	100	100	100	100	100	100	100	100	100	100	100	100	100
	END_AL	100	100	100	100	100	100	100	100	100	100	100	100	100	100	100	100
	END_KE	100	100	100	100	100	100	100	100	100	100	100	100	100	100	100	100
	HEP	100	100	100	100	100	86	25	100	100	100	100	100	100	100	100	100
	CIS_HEP	75	100	100	91	67	0	13	100	0	0	100	100	100	100	100	93
	TRANS_HEP	100	100	100	100	100	100	100	100	100	100	100	100	100	100	100	100
	MIREX	63	100	100	100	100	21	63	100	0	42	100	100	100	100	100	100

Class	Compounds	Percentage of samples with concentrations <LOQ (%)															
		**AT**	**CY**	**FI**	**FR1**	**FR2**	**IS**	**IE**	**IT**	**MT**	**NL**	**NO1**	**NO2**	**SE**	**CH**	**TR**	**UK**
Other OCPs [after 2014]	ALD	100	100	100	100	100	100	100	100	100	95	100	100	93	100	100	94
	α-CHL	0	0	0	0	0	0	0	0	0	0	0	0	0	0	0	0
	γ-CHL	0	0	0	0	0	0	6	0	0	0	0	0	0	0	0	0
	OXCHL	0	0	19	0	0	0	0	8	0	0	0	0	0	0	5	6
	DIE	36	50	94	21	0	11	4	83	13	16	33	25	20	19	58	3
	α-ENDS	55	25	94	58	50	15	100	100	25	25	17	19	33	38	26	19
	β-ENDS	73	50	100	79	63	92	100	100	40	40	89	100	93	48	63	94
	ENDS_SUL	36	13	75	37	38	8	56	67	10	0	11	19	20	10	37	13
	END	64	75	100	100	88	46	100	100	40	40	61	63	73	95	100	63
	END_AL	100	100	100	100	100	69	100	100	85	95	100	100	100	100	100	100
	END_KE	91	88	100	95	100	46	100	100	60	20	100	88	87	81	100	44
	HEP	100	63	94	58	50	23	6	67	55	10	28	69	47	38	84	25
	CIS_HEP	0	0	0	0	0	0	0	0	0	0	0	0	0	0	0	0
	TRANS_HEP	91	88	81	74	75	92	100	58	100	95	100	100	100	91	53	100
	MIREX	0	0	31	26	25	8	0	58	0	0	0	0	0	5	26	0
Dioxin-like PCBs	PCB 77	0	0	9	7	0	0	0	0	0	0	0	10	0	0	3	0
	PCB 81	0	0	27	55	8	8	0	43	0	0	17	67	13	25	31	0
	PCB 105	0	0	0	0	0	0	0	0	0	0	0	0	0	0	3	0
	PCB 114	0	0	18	28	0	0	0	0	0	0	17	24	3	17	17	0
	PCB 118	33	13	81	21	21	0	39	0	0	0	33	39	10	6	63	23
	PCB 123	0	0	18	29	0	0	0	0	0	0	0	38	3	8	24	0
	PCB 126	0	8	55	59	8	0	8	29	0	0	17	81	13	25	24	0
	PCB 156	0	0	0	14	0	0	0	0	0	0	0	5	0	0	3	0
	PCB 157	0	0	27	25	0	0	0	0	0	0	17	29	0	0	10	9
	PCB 167	0	0	9	21	0	0	0	0	0	0	0	19	0	0	7	0
	PCB 169	58	17	100	100	50	54	92	57	8	16	83	86	66	67	69	27
	PCB 189	0	0	27	54	0	0	8	14	0	0	8	76	9	17	24	0
PBDEs	BDE 28	0	0	40	13	0	6	0	0	0	0	40	19	13	0	53	0
	BDE 47	0	0	0	0	0	0	0	0	0	0	0	0	0	0	0	0
	BDE 66	0	15	87	70	25	61	0	14	30	3	92	95	53	38	90	9
	BDE 85	8	75	93	93	75	83	42	86	85	50	92	91	91	91	93	83
	BDE 99	0	0	0	7	0	0	0	0	0	0	4	0	9	0	27	0
	BDE 100	0	0	27	13	0	0	0	0	0	0	24	0	6	0	53	9
	BDE 153	8	10	80	80	50	39	17	14	30	6	84	95	53	43	83	39
	BDE 154	0	5	80	57	38	28	0	0	10	0	68	71	19	33	83	0
	BDE 183	8	0	40	37	31	28	0	29	15	3	48	81	13	14	57	0
	BDE 209	8	0	0	27	0	28	0	0	5	0	8	5	9	5	30	0

Class	Compounds	Percentage of samples with concentrations <LOQ (%)															
		AT	CY	FI	FR1	FR2	IS	IE	IT	MT	NL	NO1	NO2	SE	CH	TR	UK
HBCDD	α-HBCDD	0	0	8	0	0	19	0	0	0	0	4	0	5	0	5	0
	β-HBCDD	0	0	8	0	0	19	0	0	0	0	4	0	20	0	36	8
	γ-HBCDD	0	0	8	0	0	25	0	0	0	0	4	6	5	0	9	0
PCDD/Fs	2378-TCDD	92	50	82	90	42	62	75	100	25	19	92	91	59	27	97	18
	12378-PeCDD	83	17	82	73	17	54	25	57	8	13	92	91	22	36	90	0
	123478-HxCDD	58	25	100	80	33	62	50	100	17	6	100	95	44	55	93	18
	123678-HxCDD	42	0	100	40	0	46	42	71	8	0	100	91	25	36	97	9
	123789-HxCDD	25	25	100	70	33	46	33	86	8	13	100	95	41	46	97	9
	1234678-HpCDD	0	0	27	0	0	8	0	0	0	0	25	24	0	0	35	9
	OCDD	0	0	36	17	0	0	0	0	0	0	8	5	3	9	38	0
	2378-TCDF	8	0	46	10	0	0	0	0	0	0	17	43	0	9	10	0
	12378-PeCDF	17	0	46	30	0	0	0	0	0	0	17	33	3	9	24	0
	23478-PeCDF	8	0	46	23	0	0	0	0	0	0	25	43	3	0	10	0
	123478-HxCDF	8	0	27	20	0	15	17	0	0	3	8	33	3	0	21	0
	123678-HxCDF	8	0	36	20	0	0	0	0	0	3	25	38	6	9	24	0
	234678-HxCDF	50	17	64	43	0	23	25	43	0	3	33	67	3	18	38	9
	123789-HxCDF	67	58	91	100	100	62	100	71	50	81	92	86	91	73	90	64
	1234678-HpCDF	8	0	36	7	0	0	0	0	0	0	0	19	3	0	7	0
	1234789-HpCDF	25	33	73	90	42	62	67	57	8	19	83	76	47	73	76	18
	OCDF	0	8	27	27	8	0	25	0	0	3	8	5	6	0	28	0
**Limited Monitoring POPs**																	
	CHLODE	100	100	100	100	100	100	100	100	100	100	100	100	100	100	100	100
	PFOA	-	0	-	0	0	0	-	-	0	0	0	100	0	0	33	0
	PFOS	-	25	-	0	0	0	-	-	0	0	100	100	0	50	67	0

Table S6B. Percentage (%) of samples at each CEE MONET site in Europe that fell below the limit of quantification (LOQ) over the entire monitoring period. Site/compounds with more than 30% of samples <LOQ could not be analyzed for temporal trends and are highlighted in yellow. Values in orange depict OCP compounds with poor detection prior to changes in analytical instrumentation.

Class	Compounds	Percentage of samples with concentrations <LOQ (%)															
		BG	HR	CZ1	CZ2	CZ3	CZ4	EE	HU	LV	LT	MD	PL	RS	SK	SI	UA
Indicator PCBs	PCB 28	0	0	2	1	2	0	0	0	0	5	0	0	0	0	0	0
	PCB 52	0	0	2	1	2	0	0	0	0	5	0	0	0	0	0	0
	PCB 101	3	3	0	1	2	0	17	7	0	16	0	0	0	0	18	0
	PCB 138	26	45	3	9	2	0	29	10	25	23	0	0	9	21	35	0
	PCB 153	14	3	2	1	2	0	23	3	8	24	0	0	0	0	28	0
	PCB 180	39	48	17	16	3	2	0	50	44	46	15	41	14	48	41	4
OCPs	PeCB	0	0	2	1	2	0	0	0	0	0	0	0	0	0	0	0
	HCB	0	0	0	0	0	0	0	0	0	0	0	0	0	0	0	0
	α-HCH	0	0	0	1	2	0	0	0	0	5	0	0	0	0	0	0
	β-HCH	26	35	30	19	20	9	38	6	24	24	0	3	12	27	63	0
	γ-HCH	5	0	0	1	2	0	0	0	0	0	0	0	0	0	0	0
	δ-HCH	67	41	13	7	13	0	13	38	53	50	0	49	32	5	56	0
	o,p'-DDD	37	52	27	9	7	4	0	22	38	30	0	0	12	17	53	0
	p,p'-DDD	41	48	29	5	2	3	29	27	36	35	0	0	12	20	40	0
	o,p'-DDE	12	35	12	7	7	0	6	0	27	25	0	0	0	4	31	0
	p,p'-DDE	0	0	0	0	0	0	0	0	0	0	0	0	0	0	0	0
	o,p'-DDT	6	10	0	0	0	2	0	0	3	3	0	0	0	8	16	0
	p,p'-DDT	6	10	9	3	3	0	12	3	6	16	0	0	0	17	14	0
Other OCPs [before 2014]	ALD	100	25	100	100	100	100	0	100	100	100	100	100	100	100	100	100
	α-CHL	91	100	94	100	100	47	0	92	100	100	86	83	88	100	100	42
	γ-CHL	100	100	94	100	100	88	0	100	100	100	86	92	81	100	100	50
	OXCHL	100	100	100	100	100	100	0	100	100	100	100	100	100	100	100	100
	DIE	100	100	94	100	100	59	0	85	100	100	100	92	44	100	100	33
	α-ENDS	9	25	65	59	71	29	0	31	83	92	57	17	19	67	42	17
	β-ENDS	100	100	100	100	100	100	0	100	100	100	100	100	100	100	75	100
	ENDS_SUL	100	100	100	100	100	100	0	100	100	100	100	100	100	100	100	100
	END	100	100	100	100	100	100	0	100	100	100	100	100	100	100	100	100
	END_AL	100	100	100	100	100	100	0	100	100	100	100	100	100	100	100	100
	END_KE	100	100	100	100	100	100	0	100	100	100	100	100	100	100	100	100
	HEP	100	0	100	100	100	100	0	100	100	100	100	100	100	100	100	100
	CIS_HEP	100	100	94	100	100	77	0	100	100	100	100	100	100	100	100	75
	TRANS_HEP	100	100	100	100	100	100	0	100	100	100	100	100	100	100	100	100
	MIREX	100	100	100	100	100	100	0	100	100	100	100	100	100	100	100	100

Class	Compounds	Percentage of samples with concentrations <LOQ (%)															
		BG	HR	CZ1	CZ2	CZ3	CZ4	EE	HU	LV	LT	MD	PL	RS	SK	SI	UA
Other OCPs [after 2014]	ALD	90	8	100	100	100	100	100	100	100	100	100	95	100	100	100	79
	α-CHL	5	0	0	0	0	0	100	0	0	10	0	0	0	0	0	0
	γ-CHL	5	0	0	0	0	0	100	0	4	10	0	0	0	0	0	0
	OXCHL	5	8	0	5	5	0	100	0	4	15	0	0	13	0	5	14
	DIE	5	0	22	26	32	35	100	0	33	55	75	40	31	0	55	36
	α-ENDS	15	0	33	37	37	35	100	0	29	50	100	25	38	0	15	21
	β-ENDS	70	17	72	63	79	53	100	15	96	90	100	45	56	50	25	57
	ENDS_SUL	5	0	0	16	16	0	100	8	33	25	100	0	6	0	0	0
	END	80	25	94	84	74	71	100	100	92	95	100	80	88	100	100	79
	END_AL	100	100	100	100	100	100	100	100	100	100	100	95	100	100	100	100
	END_KE	90	63	89	95	100	88	100	85	88	95	100	80	100	100	100	100
	HEP	10	4	56	58	42	41	100	46	75	70	75	60	19	90	60	0
	CIS_HEP	5	8	0	0	0	0	100	0	4	10	0	5	0	0	0	0
	TRANS_HEP	100	25	83	95	84	82	100	100	96	100	75	100	100	100	100	100
	MIREX	5	13	0	0	11	0	100	0	0	10	100	0	0	0	5	0
Dioxin-like PCBs	PCB 77	0	0	3	6	0	0	0	0	0	0	0	0	0	0	0	0
	PCB 81	0	0	24	11	14	0	0	0	17	6	43	0	3	0	0	17
	PCB 105	0	0	3	0	0	0	0	0	0	0	0	0	0	0	0	0
	PCB 114	0	0	12	11	8	0	0	0	3	0	0	0	3	0	0	0
	PCB 118	41	23	9	14	0	0	40	19	13	27	0	0	0	16	55	0
	PCB 123	0	0	12	11	6	3	0	0	7	0	0	0	0	0	8	8
	PCB 126	0	0	32	19	11	3	0	15	17	0	43	0	16	0	17	17
	PCB 156	0	0	3	8	0	0	0	0	0	0	0	0	0	0	0	0
	PCB 157	0	0	15	11	8	0	0	4	3	0	0	0	3	0	0	0
	PCB 167	8	0	3	8	0	0	0	0	0	0	0	0	0	0	0	0
	PCB 169	50	0	68	42	42	39	36	27	53	25	57	8	36	68	100	33
	PCB 189	8	0	21	11	8	3	18	8	27	0	57	0	3	0	8	8
PBDEs	BDE 28	14	0	21	6	0	6	27	8	30	7	0	4	3	32	8	0
	BDE 47	0	0	0	0	0	0	0	0	0	4	0	0	0	11	0	0
	BDE 66	64	65	71	50	47	41	73	50	63	50	9	29	16	63	46	33
	BDE 85	82	70	94	89	89	82	91	96	80	93	100	100	84	100	100	91
	BDE 99	7	0	3	0	0	3	0	0	10	4	0	0	0	16	4	0
	BDE 100	18	0	15	0	0	3	18	15	27	21	0	8	3	42	17	14
	BDE 153	57	60	77	67	42	47	64	50	63	61	46	29	38	74	75	29
	BDE 154	46	30	68	44	31	27	46	19	60	46	9	4	13	63	63	19
	BDE 183	39	50	41	33	14	32	18	8	57	29	18	13	19	58	29	14
	BDE 209	18	5	6	14	11	18	0	23	30	29	9	13	22	53	21	5

Class	Compounds	Percentage of samples with concentrations <LOQ (%)															
		BG	HR	CZ1	CZ2	CZ3	CZ4	EE	HU	LV	LT	MD	PL	RS	SK	SI	UA
HBCDD	α-HBCDD	0	26	32	27	8	31	25	0	0	0	0	6	0	0	6	8
	β-HBCDD	0	26	39	35	31	39	25	0	15	6	0	25	0	0	6	8
	γ-HBCDD	0	26	25	23	0	15	0	0	0	0	0	0	0	0	0	8
PCDD/Fs	2378-TCDD	83	38	74	57	33	28	55	40	50	25	29	17	56	37	82	25
	12378-PeCDD	67	13	46	29	17	8	36	24	43	25	29	8	31	11	82	8
	123478-HxCDD	92	88	74	37	19	14	55	24	53	44	29	17	38	58	82	25
	123678-HxCDD	25	25	54	26	3	3	36	8	30	25	14	8	22	26	55	8
	123789-HxCDD	50	25	69	40	19	17	55	20	60	31	43	17	28	42	64	25
	1234678-HpCDD	8	0	6	3	0	0	0	0	3	6	0	0	0	0	0	0
	OCDD	0	0	6	0	0	0	9	0	3	0	0	0	13	5	0	0
	2378-TCDF	0	0	3	3	0	0	0	0	0	0	0	0	3	0	0	0
	12378-PeCDF	0	0	11	3	0	0	0	0	0	0	0	0	0	0	18	0
	23478-PeCDF	8	0	9	0	0	0	0	0	0	0	0	0	0	0	0	0
	123478-HxCDF	8	0	17	0	0	0	9	0	3	0	0	0	0	5	9	0
	123678-HxCDF	0	0	20	0	0	0	0	0	3	0	0	0	3	5	9	0
	234678-HxCDF	8	0	29	6	0	6	9	0	7	0	29	0	6	0	27	0
	123789-HxCDF	75	100	91	66	36	50	64	48	73	63	57	58	50	74	73	33
	1234678-HpCDF	0	0	3	0	0	0	0	0	3	0	0	0	3	0	9	0
	1234789-HpCDF	42	0	57	23	17	19	27	16	27	13	43	8	22	58	64	17
	OCDF	0	0	17	6	3	6	18	4	10	0	0	0	13	0	18	8
**Limited Monitoring POPs**																	
	CHLODE	100	100	100	100	93	100	100	100	100	100	100	100	100	100	100	100
	PFOA	0	-	29	29	14	57	25	50	-	0	-	0	13	-	0	0
	PFOS	0	-	29	0	0	14	50	50	-	0	-	0	13	-	75	0

3. TREND ANALYSIS

The length of temporal trends vary depending on the compound and when each site was established, with most sites in CEE established earlier than those in WEOG (Figure S1). The longest trends included in this study are at Košetice (15.8 years, *n* = 69 samples) and Prague Libuš (15.0 years, *n* = 66), which were established in 2003 and 2004, respectively, during the inception of the national Czech MONET-CZ network. Of the sites monitored during the concurrent APOPSBAL campaign in 2004, only Fruška Gora (14.4 years, *n* = 45) and Zagreb (14.4 years, *n* = 31) have continued long-term monitoring under MONET. Most of the other sites in CEE began monitoring in 2006/2007, with trends ranging in length from 9.1 years at Lahemaa (n = 26) to 13.0 years at Churáňov (n = 61). The remaining sites included in this study were all established in 2009, with trends ranging in length from 7.6 years at Sonnblick (n = 28) to 9.8 years at Moussala (n = 40).

Routine monitoring of PBDEs, dl-PCBs, and PCDD/Fs began in 2011 at all sites so the temporal trends for these compounds are shorter than those of the other POPs, but more similar in length between sites, ranging from 6.5 years at Pallas (n = 15) to 8.1 years at Košetice (n = 36). Monitoring of HBCDDs began in 2012/2013, thus sampling durations are even shorter, ranging from 4.8 years at Çamkoru (n = 22) to 7.0 years at Zeppelin (n = 18).

Due to poor analytical detection of the 'Other OCPs' (aldrin, chlordane, dieldrin, endosulfan, endrin, heptachlor, mirex) during the initial years of MONET, data series for these compounds began in 2014/2015 at most sites, corresponding to changes in analytical instrumentation resulting in improved detection (Table S6). Data series for these compounds are therefore shortest, with all below 5 years aside from Rucava and Zagreb (both 5.3 years, n = 24). The one exception is dieldrin, for which elevated concentrations were detected at De Zilk, Ġordan Lighthouse, High Muffles, Mace Head, and Stórhöfði in the initial years of monitoring, resulting in data series that are up to 5.6–7.5 years in length.

In total, nearly 3,000 trend plots were generated, representing each site/compound combination. To simplify visualization of the trends in the Supporting Information, we have provided one example of a trend plot with all data points, the calculated trendline, and its confidence intervals (Figure S2). Additional plots showing the combination of all trendlines (no data points or confidence intervals to avoid clutter) for each major POP group for sites in both WEOG and CEE countries are provided in Figure S3.

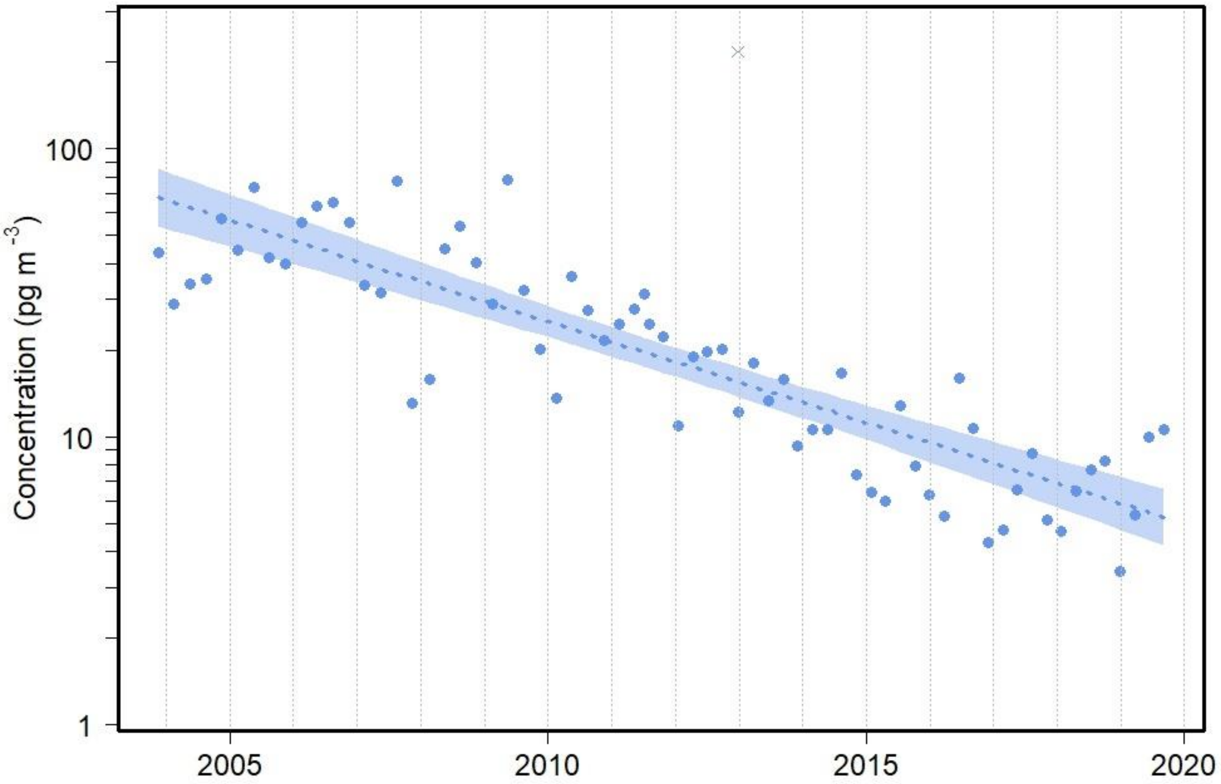

Figure S2. Example trend plot for γ-HCH at Košetice. Each data point represents the concentration measured in a single 84-d PUF sample with a total of 68 measurements over the monitoring period from 2003–2019. The dashed line is the calculated trend and the shaded area is its 95% confidence interval.

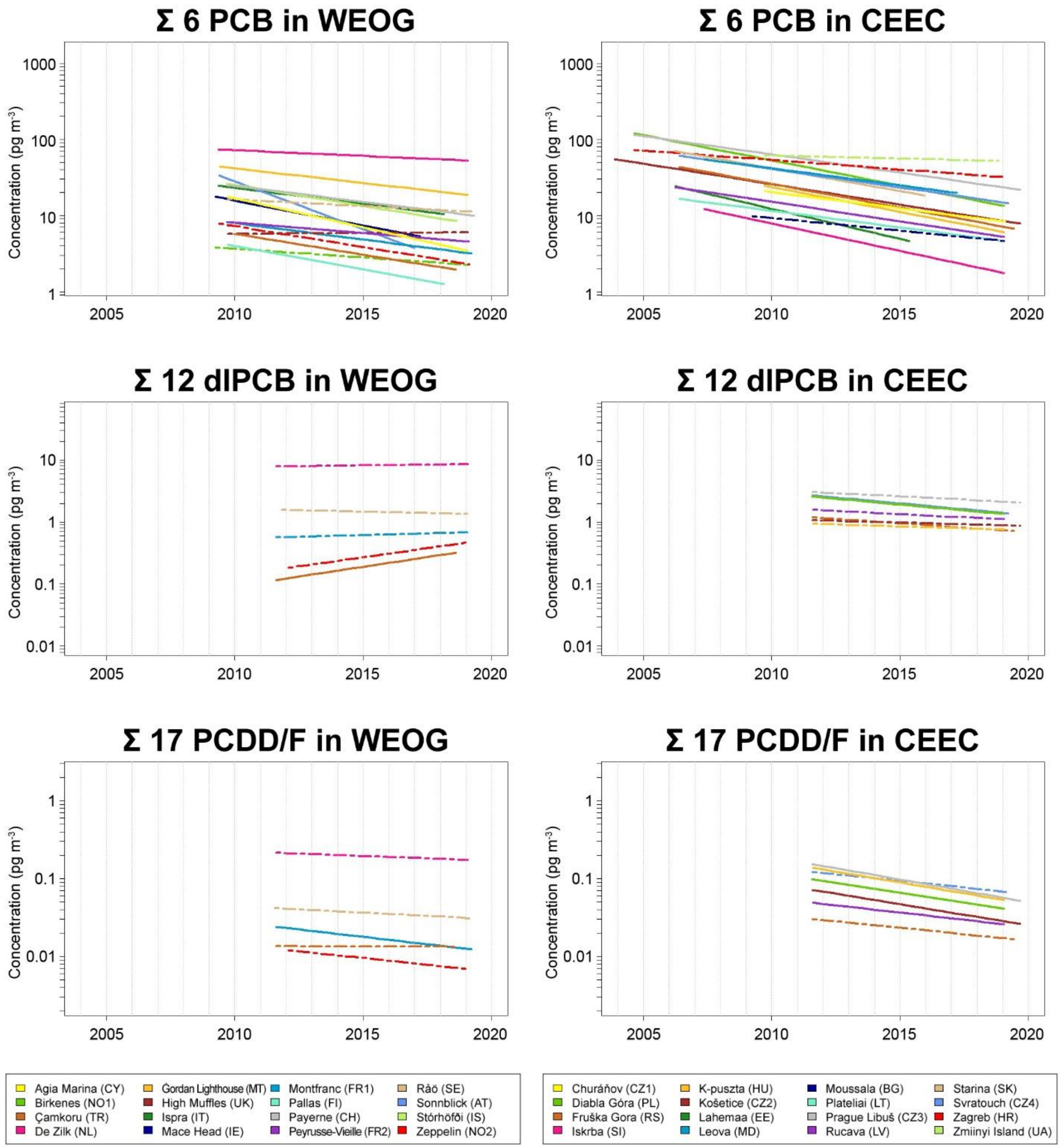

Figure S3A. Temporal trends for $\sum_6$PCB, $\sum_{12}$dl-PCB, and $\sum_{17}$PCDD/F at MONET sites in WEOG and CEE countries. Solid lines depict statistically significant trends; dashed lines depict statistically insignificant trends.

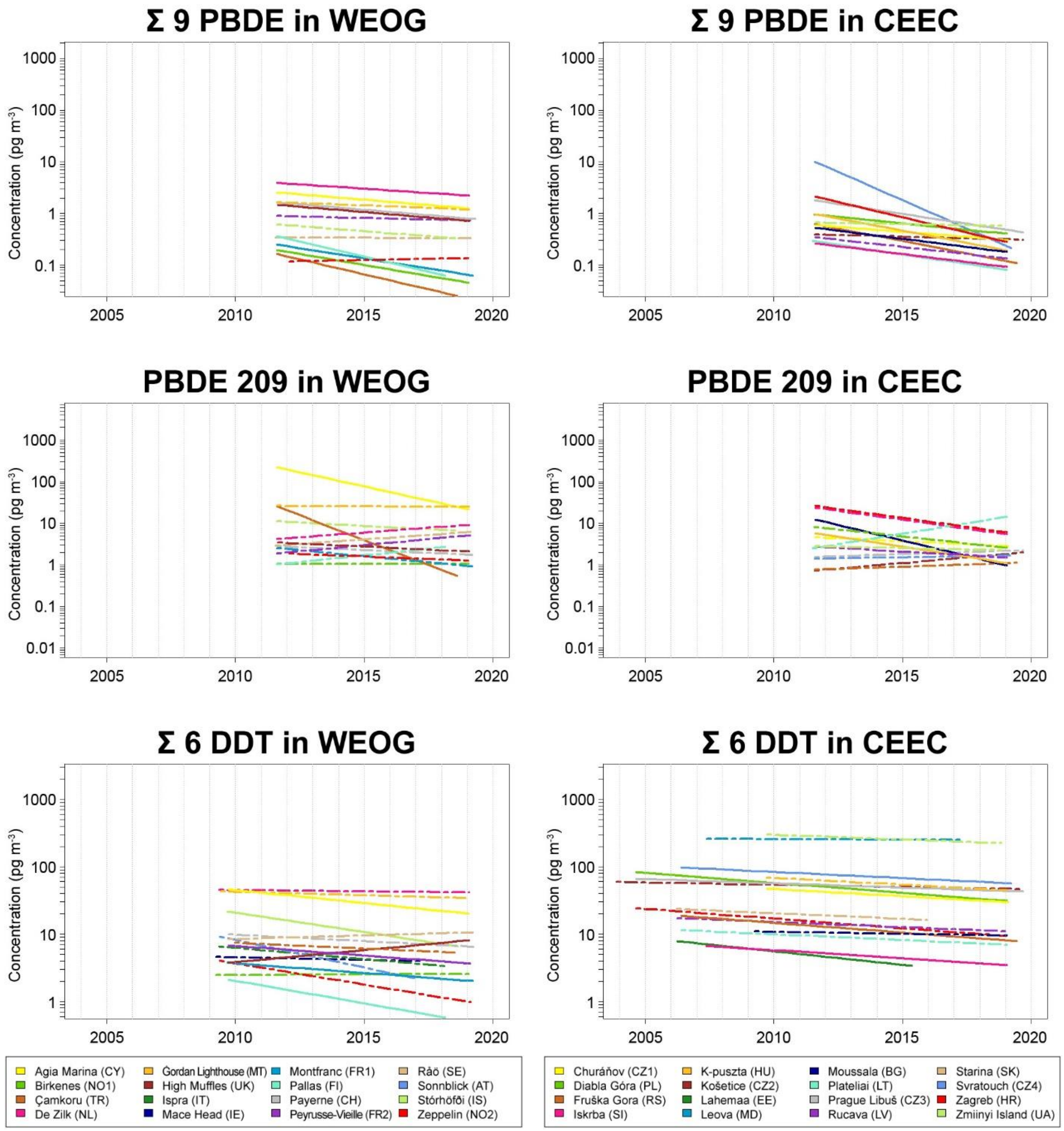

Figure S3B. Temporal trends for $\sum_9$PBDE, BDE 209, and $\sum_6$DDT at MONET sites in WEOG and CEE countries. Solid lines depict statistically significant trends; dashed lines depict statistically insignificant trends.

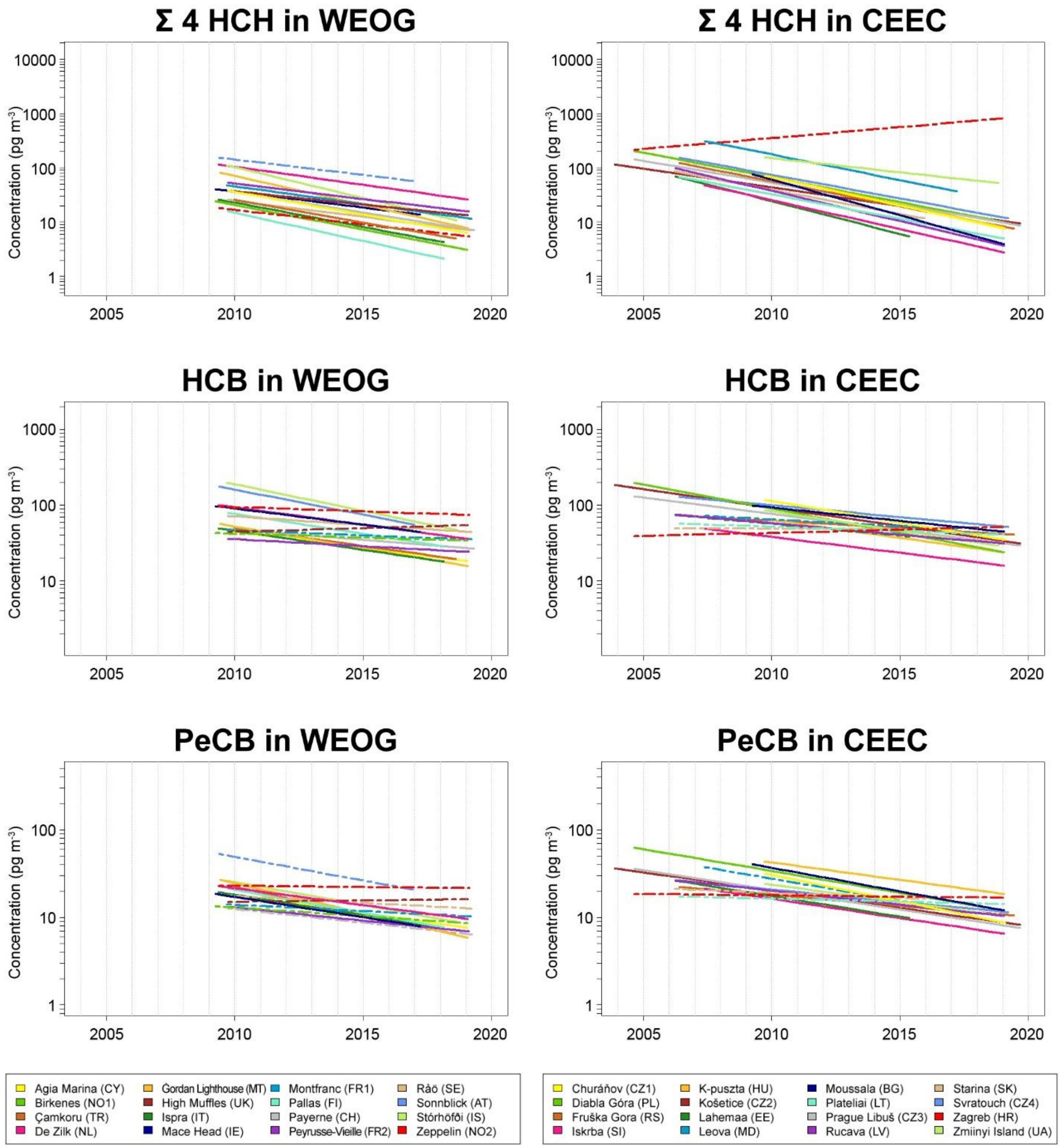

Figure S3C. Temporal trends for $\sum_4$HCH, HCB, and PeCB at MONET sites in WEOG and CEE countries. Solid lines depict statistically significant trends; dashed lines depict statistically insignificant trends.

4. SPATIAL ANALYSIS

4.1. Continental Transects

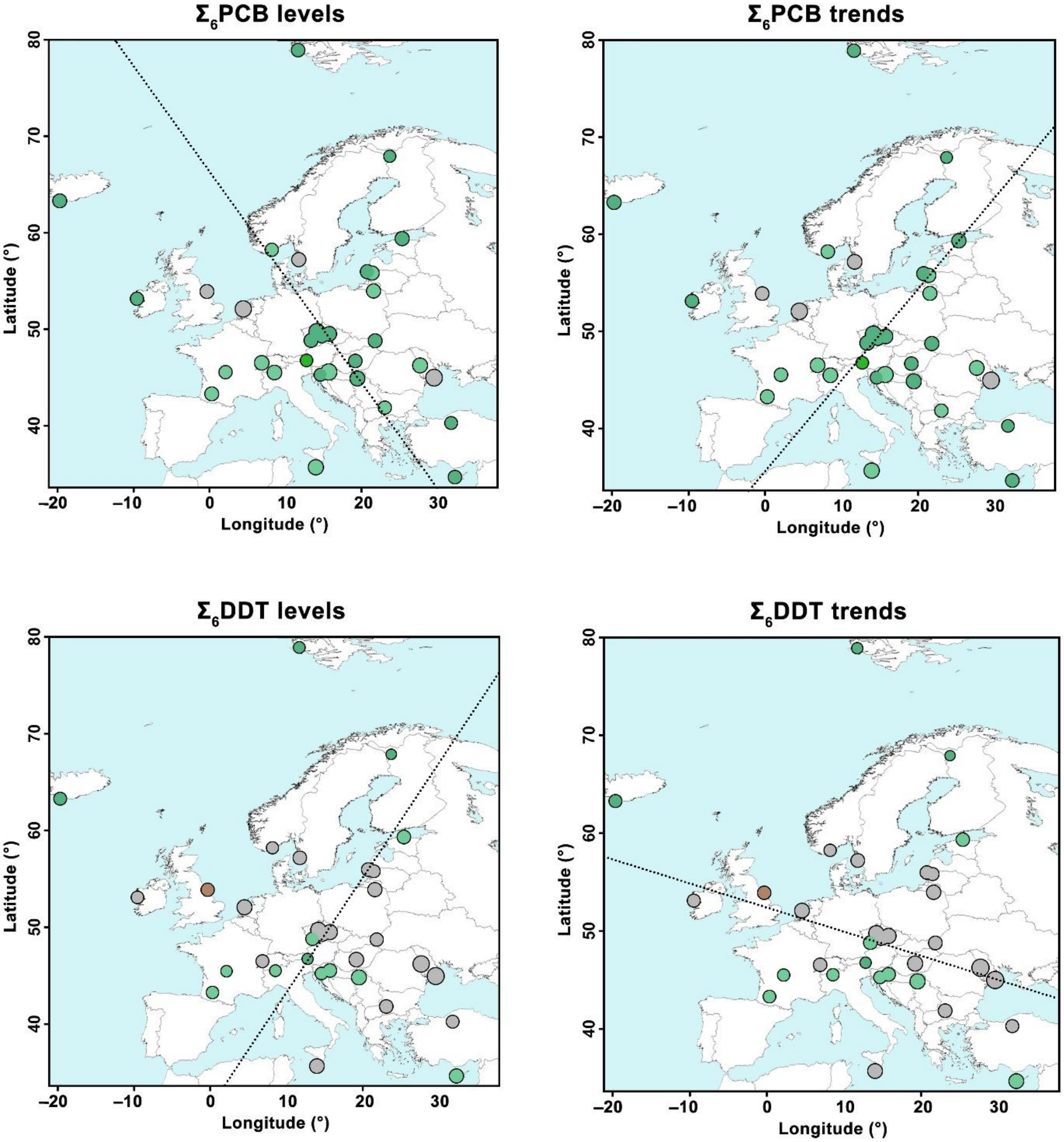

Figure S4A. Transects dividing sites across the continent into the two most different halves based on the concentrations and trends of $\sum_6$PCB and $\sum_4$DDT. Sizes of circles represent the relative concentrations at each site (small is low, large is high). Colours of circles represent the direction of the trend at each site (red is increasing trend, grey is flat trend, green is decreasing trend).

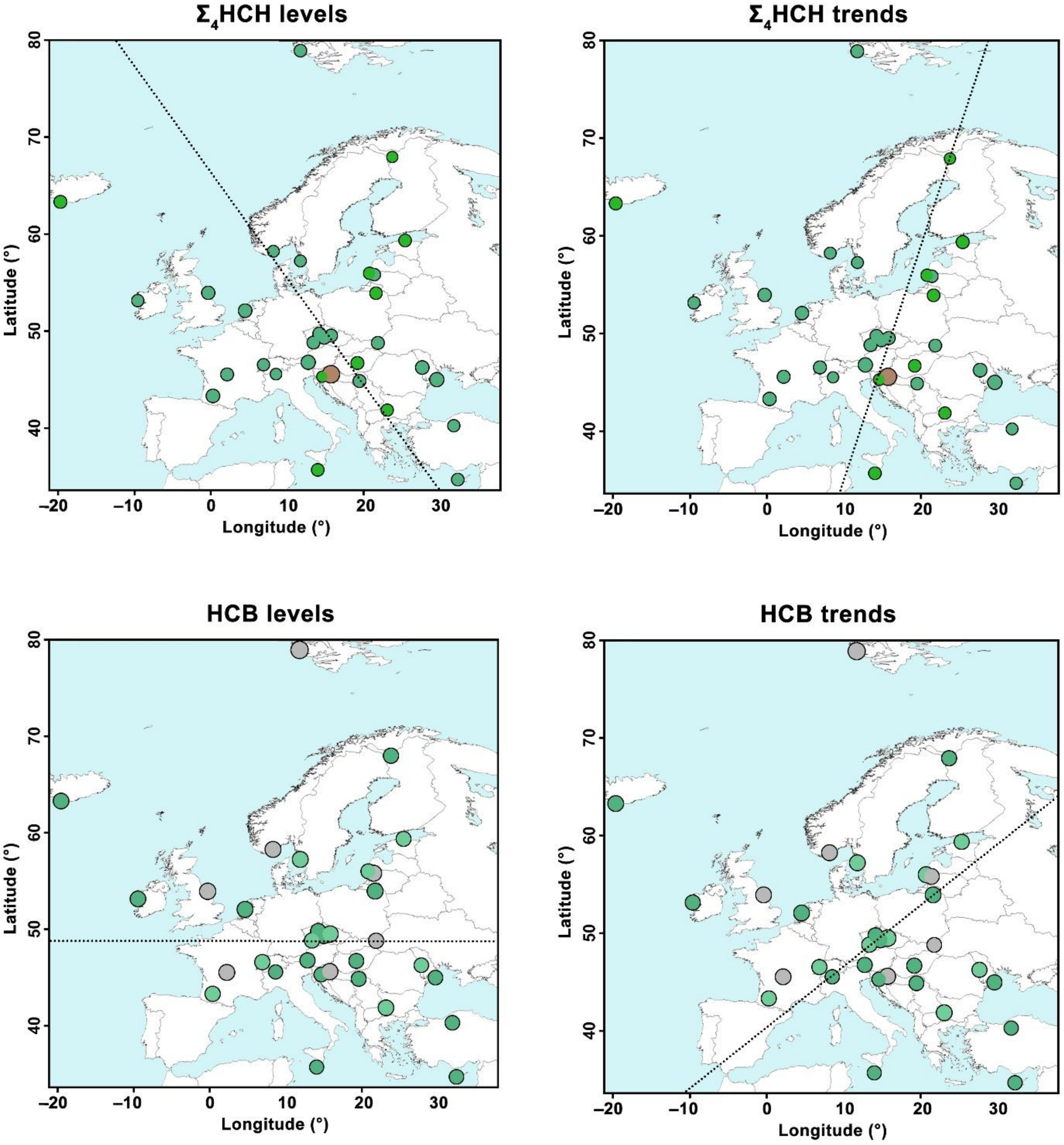

Figure S4B. Transects dividing sites across the continent into the two most different halves based on the concentrations and trends of $\sum_4$HCH and HCB. Sizes of circles represent the relative concentrations at each site (small is low, large is high). Colours of circles represent the direction of the trend at each site (red is increasing trend, grey is flat trend, green is decreasing trend).

4.2. Cluster Analysis

Table S7. Site characteristics and log-transformed trend and concentration input parameters for the multidimensional cluster analysis.

Site Name (Code)	Latitude	Logitude	Elevation (m asl)	Median wind speed (m/s)	$\sum_6$PCB Conc.	$\sum_6$DDT Conc.	$\sum_4$HCH Conc.	HCB Conc.	$\sum_6$PCB Trend	$\sum_6$DDT Trend	$\sum_4$HCH Trend	HCB Trend
Agia Marina (CY)	35.038	33.058	532	2.8	-0.7	0.5	-0.5	-1.7	-1.3	-0.8	-0.2	-0.6
Çamkoru (TR)	40.585	32.505	1406	1.9	-1.3	-0.5	-0.8	-1.5	-0.3	0.2	-0.2	-0.8
Ispra (IT)	45.817	8.633	209	1.1	0.3	-0.9	-1.2	-1.3	0.1	-0.4	-0.4	-0.8
Mace Head (IE)	53.330	-9.900	5	5.4	-0.6	-0.8	-0.4	-0.1	-0.7	0.6	0.6	-0.6
Pallas (FI)	68.000	24.246	340	0.6	-1.9	-2.1	-1.6	0.0	-0.5	-1.8	-0.7	-0.8
Sonnblick (AT)	47.054	12.958	3110	0.4	-1.6	-1.8	1.5	-0.6	-2.9	-2.4	0.6	-1.4
Stórhöfði (IS)	63.400	-20.283	118	5.2	0.1	-0.4	0.2	0.6	-0.3	-1.4	-1.0	-1.6
Iskrba (SI)	45.561	14.863	520	0.2	-1.4	-0.7	-1.2	-1.6	-0.9	-0.2	-0.7	-0.4
Lahemaa (EE)	59.515	25.928	61	4.6	-0.1	-0.1	-0.1	0.0	-1.3	-0.8	-1.3	0.5
Birkenes (NO1)	58.383	8.250	190	0.5	-1.0	-0.9	-0.8	0.7	0.7	1.2	-0.2	1.1
High Muffles (UK)	54.140	-0.460	270	4.7	-0.3	-0.2	0.4	1.1	2.4	2.6	1.0	2.1
Montfranc (FR1)	45.810	2.060	810	2.6	-0.8	-1.1	0.2	0.5	0.3	-0.2	0.6	1.1
Payerne	46.800	6.933	489	1.8	0.5	-0.3	-0.1	-0.1	0.3	0.2	0.3	0.5
Peyrusse-Vieille (FR2)	43.630	0.180	175	1.6	-0.4	-0.6	0.6	-0.3	0.7	-0.4	0.7	0.5
Råö (SE)	57.394	11.914	10	2.8	0.3	-0.1	-0.4	0.6	1.4	1.6	0.7	0.5
Zeppelin (NO2)	78.880	11.883	474	3.5	-0.7	-1.3	0.0	2.8	-0.3	-1.8	0.9	1.3
Churáňov (CZ1)	49.068	13.615	1121	1.8	0.1	-0.1	-0.1	0.3	-0.7	-0.2	-0.4	0.7
Moussala (BG)	42.179	23.585	2925	0.6	-0.5	-0.1	-0.5	0.9	0.7	0.6	-1.5	0.0
Plateliai (LT)	56.010	21.887	150	2.5	-0.4	-0.4	-0.6	0.4	0.1	0.2	-0.2	1.1
Rucava (LV)	56.162	21.173	16	3.6	-0.3	0.0	-0.9	-0.1	-0.1	0.4	-0.8	0.3
Starina (SK)	49.043	22.260	345	0.9	-0.1	-0.1	-0.1	0.0	-0.7	0.2	-0.4	1.3
De Zilk (NL)	52.297	4.5110	-	5.3	2.1	0.9	0.9	0.5	1.4	0.8	0.4	-0.6
Ġordan Lighthouse (MT)	36.073	14.219	167	4.4	1.0	0.8	-0.1	-2.1	0.3	0.6	-0.8	-1.2
Diabla Góra (PL)	54.125	22.038	157	2.9	0.1	0.7	-0.3	0.4	0.1	0.0	-0.7	-0.8
Fruška Gora (RS)	45.159	19.863	514	1.7	0.7	0.8	0.1	-0.7	-0.9	-0.4	-0.4	-1.4
Košetice (CZ2)	49.573	15.080	503	2.7	0.3	1.1	-0.1	0.5	-0.3	0.8	0.4	-0.6
K-puszta (HU)	46.968	19.553	-	2.1	-0.2	1.0	-0.2	-0.9	-0.7	0.0	-0.7	-0.6
Leova (MD)	46.500	28.300	154	2.4	0.8	2.1	0.8	0.0	0.1	1.0	-0.4	0.7
Prague Libuš (CZ3)	50.007	14.446	302	2.4	1.4	1.1	-0.1	0.1	-0.1	0.4	-0.1	-0.6
Svratouch (CZ4)	49.735	16.034	735	2.8	0.8	1.2	0.2	0.9	-0.1	0.2	-0.2	0.0
Zmiinyi Island (UA)	45.256	30.201	28	4.6	2.2	2.2	1.6	-0.6	1.8	0.4	0.9	-1.0
Zagreb (HR)	45.836	15.983	408	1.5	1.6	0.1	3.9	1.1	1.0	-0.4	4.1	2.1

6. REFERENCES

(1) Pribylova, P.; Kares, R.; Boruvkova, J.; Cupr, P.; Prokes, R.; Kohoutek, J.; Holoubek, I.; Klanova, J. Levels of Persistent Organic Pollutants and Polycyclic Aromatic Hydrocarbons in Ambient Air of Central and Eastern Europe. *Atmos. Pollut. Res.* **2012**, *3* (4), 494–505. https://doi.org/10.5094/APR.2012.057.

(2) UNEP. Stockholm Convention Global Monitoring Plan for Persistent Organic Pollutants: First Global Monitoring Report. *UNEP/POPS/COP.4/33* **2009**, 1–20.

(3) UNEP. Stockholm Convention Global Monitoring Plan for Persistent Organic Pollutants: Second Global Monitoring Report. *UNEP/POPS/COP.8/INF/38* **2017**, 1–125.

(4) Kalina, J.; Scheringer, M.; Borůvková, J.; Kukučka, P.; Přibylová, P.; Bohlin-Nizzetto, P.; Klánová, J. Passive Air Samplers As a Tool for Assessing Long-Term Trends in Atmospheric Concentrations of Semivolatile Organic Compounds. *Environ. Sci. Technol.* **2017**, *51* (12), 7047–7054. https://doi.org/10.1021/acs.est.7b02319.
